# Skin disease diagnostics through federated transfer learning on heterogeneous data

**DOI:** 10.1038/s41598-025-31730-7

**Published:** 2026-01-15

**Authors:** Shikha Sharma, Ruchi Mittal, Nitin Goyal, S. B. Goyal, Chaman Verma

**Affiliations:** 1 Chitkara University Institute of Engineering and Technology, Chitkara University, Rajpura, Punjab India; 2 Department of Computer Science and Engineering, School of Engineering and Technology, , Central University of Haryana, Mahendragarh, Haryana India; 3 Department of Media and Educational Informatics, Faculty of Informatics, Eötvös Loránd University, Budapest, 1053 Hungary

**Keywords:** Skin disease, Federated learning, Transfer learning, Dense neural network, Feature extraction, Classification, Biotechnology, Cancer, Computational biology and bioinformatics

## Abstract

Skin diseases frequently cause mental and physical distress and are major global health concern. Because early detection is crucial to successful treatment, accurate diagnosis is challenge for dermatologists as well. Diagnostic accuracy could be significantly enhanced using methods like machine learning (ML) and deep learning (DL). However, substantial datasets are required for these models to make accurate predictions. The healthcare providers frequently encounter data shortages, and privacy regulations restrict data sharing. A privacy-preserving federated transfer learning for diagnosing skin diseases which incorporate four key strategies to enhance effectiveness. The transfer learning is used to train a model with dense neural network (DNN) for skin diseases detection. The feature extraction is performed using pre-trained architectures and DNN is used for classification. The federated learning (FL) replaces the transfer learning to train the model across distributed nodes with the DNN used to disease detection. The FL is combined with transfer learning to build a cohesive ecosystem where data privacy is maintained. The model performance was validated on both IID and non-IID database, with the proposed feature extraction with federated learning model achieving cross validation accuracy of 99.528% and 99.689% for IID and non-IID database, respectively. Results indicate that feature extraction with FL model can produce efficient, lightweight models—well-suited for resource-constrained devices—while ensemble learning enhances edge device performance, offering a powerful and privacy-preserving solution for skin disease diagnosis in modern healthcare.

## Introduction

Globally, millions of people of all ages and demographics suffer from skin problems. Skin ailments range from eczema, psoriasis, and acne to melanoma and other skin malignancies^1^. Chronic illnesses like psoriasis can cause physical discomfort, emotional suffering, and social isolation^2^. Non-fatal skin diseases account for a large portion of global healthcare costs. The dermatologist scarcity in many places delays diagnoses and worsens patient outcomes^3^. Skin illnesses can indicate underlying health difficulties, thus early and precise diagnosis is crucial to preserving patient health and possibly detecting additional systemic diseases^4^. Dermatologists directly examine lesions, pigmentation, and texture changes to diagnose skin illnesses^5,6^. Analyzing large datasets of skin images and finding disease patterns with artificial intelligent (AI) based techniques is also improving diagnostic accuracy^7^. Despite technological advances, such equipment and technical competence are scarce, especially in low-resource areas^8^. In dermatology, virtual and real-time skin condition diagnosis are now possible through advanced digital tools^9,10^. Patients benefit from quick assessments and teledermatology consultation improves the dermatological care accessibility^10^. Continuous observation allows for personalized treatment adjustments, improving patient outcomes and adherence^11^. Additionally, AI models can analyze patient data to detect early skin abnormalities and potentially identify skin cancers or other serious conditions^11,12^. However, as these digital healthcare ecosystems expand, concerns about data security and privacy become increasingly significant, particularly in dermatology where sensitive medical data is transmitted and stored^12^.

Medical imaging and diagnosis capture and share sensitive health data across platforms, making data privacy as serious problem^13^. Medical images used in dermatology contain visual data about skin problems and information that could reveal identification of patients if privacy protections are insufficient. Centralized storage systems, which contain patient data from numerous sources, are particularly vulnerable to hackers, threatening patient privacy and confidence in digital health care systems^14^. Federated learning (FL) model allows decentralized data utilization on local devices while keeping it secure, allowing shared model advances without transferring patient data^15^. To prevent data leaks during training, FL modelrequires strong encryption and secure aggregation. These advances make it harder to balance data utility and privacy since models need enough data to be clinically useful without violating patient privacy^16^. FL and transfer learning models have been popular in medical application because they solve data privacy, limited resources, and model adaptability^17^. FLmodel makes it possible to train machine learning (ML) and deep learning (DL) models on dispersed datasets, such as medical servers, without the need for centralized collection^18^. Transfer learning model allows pre-trained models on huge, publically available datasets to be tailored to specific medical applications with less task-specific data^19^. Transfer learning lets models adapt to diverse healthcare domains, such as dermatology and radiology. Transfer learning along with FL, can improve medical diagnostic accuracy by using information from many data sources, even in resource-limited medical environments^20^. These methods promise to improve model performance while protecting privacy and managing data scarcity, enabling ethical and practical AI use in healthcare. FL models with decentralized data interested by the discretion subjects of traditional ML/DL techniques that have been previously discussed. After that, each local network model is trained using its own local data, preventing sensitive information from being shared over a server network. The rest of this paper is organized as follows. The literature on skin disease diagnostic using ML/DL techniques is reviewed in Section "Related Work". The proposed model for diagnosing skin diseases using transfer learning, pre-trained feature extraction models, federated feature extraction, and federated transfer learning is presented in Section "Methodology skin disease diagnosis". The experimental setup and results comparison of skin disease detection models are described in Section "Results analysis" and "Discussion". The paper conclusion and future scope discussed in Section "Conclusion".

## Related work

By handling visual complexity and model generalization through image augmentation, the convolutional neural network (CNN) offers a diverse dataset that more accurately captures the variability of skin conditions^21^. The model’s accuracy of 86% and reminiscence of 81% across seven disease classes show that it can recognize the features of skin disorders. The FL framework^22^ aggregates prediction while sharing sensitive data. FL differential privacy architecture facilitates cooperative model training without transferring confidential patient data to central servers using decentralized manner^23^. The implementation is on Amazon’s AWS cloud system, showed ease of use and scalability^24^ which improves mobile health technology diagnostics. A hybrid model using CNN and optimization module^25^ is used to improve the gesture identification. FL pre-trains the mixed approach without revealing sensitive sEMG data, and then transfer learning fine-tunes the model for each subject based on their features. According to experimental results, this approach improves recognition accuracy by 12.01% over conventional FL model and 28.52% over local training, overcoming data shortage and prioritizing privacy. The FL is used to train global model and sharing encrypted parameters via blockchain with permission to address privacy and trust issues^26^. According to the data, the scheme outperforms baseline models in segmentation by 19.08% in Hausdorff distance for whole malignancies and 1.99% in Dice comparison coefficient for attractive growths. The local devices run simulations on their datasets without transferring sensitive health data, solving privacy concerns^27^. Radar-based heartbeat and activity monitoring is implemented using a networked multi-task transfer learning^28^. FedRadar beats local training models in heartbeat rate prediction and action planning on actual radar datasets by 2.8% and 2.5%, respectively. FL with decentralized data storage improves the detection rate^29^. A data balancing strategy improves classifier performance and achieves 95% accuracy by correct the dataset’s class imbalance. FRESH is smart healthcare architecture that combines FL with ring identity safeguards against such assaults^30^. Modified batch verification takes advantage of lined operations’ additively on elliptic arches to ease the server’s dispensation load.

### Review summary

Based on the literature review (Table 1), DL techniques used to draw attention to the problems of using FL for skin disease diagnosis^21–30^. The inherent non-IID distribution and data imbalance in skin disease datasets are significant issues. Patients from various demographic groups, geographical locations, and healthcare facilities have varying disease frequencies and image features, which leads to biased models that are not particularly successful at generalizing to other populations. Threats to security and privacy are another significant obstacle. In a medical context, protecting patient information’s security and confidentiality is essential. The FL system^31^, which uses a dataset of over 10,000 photos and decentralized data, initially demonstrates an overall accuracy rate of around 79% in the classification of skin disorders. The four categories of skin diseases are classified using the CNN^32^ and the parameters are optimized using the hyper-parameter tuning.Even though FL is decentralized, during model updates, sensitive patient data—such as images of skin lesions—is still susceptible to reconstruction or inference assaults. The varied nature of medical imaging data, which unintentionally expose distinguishable characteristics, increases this danger^21,23^.

**Table 1:** Research gap summary from existing FLfor disease diagnosis frameworks.

References	Disease	Technique	Dataset	Training place	Findings	Research gaps
^21^	Skin disease detection	FL with CNN	Synthetic dataset	Decentralized	Accuracy 82.42%	The performance is not further explored in larger datasets
^22^	Malaria image detection	FL with ResNet-50 and DenseNet	27,560 images	Decentralized	Accuracy 92%, 72%	Hyper tuning could be enhanced with use of some optimizer
^23^	Detect Tuberculosis	FL with CNN	Chest X-ray dataset	Decentralized	Accuracy 89.56%	Higher time consumption in training dataset
^24^	Seizure epilepsy monitoring	FL with ResNet and Transformer	CHB-MIT dataset	Decentralized	Accuracy 88.4%	Not consider the dataset overfitting problems
^25^	sEMGhand gesture recognition	Federated transfer learning	Ninapro DB5 dataset	Decentralized	Accuracy 87.96%	Real-time could be challenging, requires standard procedures
^26^	Brain tumour segmentation	FL with CNN and Blockchain	BRAST dataset	Decentralized	Dice 92.35%	Privacy and data utilityleads to degradation of prediction results
^27^	Mental stress detection	FL with SVM	Synthetic dataset	Decentralized	Communication overhead 10.02 MB/day	Loss of essential image with edges which causes ineffective classification
^28^	Heartbeat rate activity classification	FL with multi-task neural network	Kaggle dataset	Decentralized	Accuracy 93%	Not considering the resource-constraints and complexity
^29^	Skin disease classification	FL with CNN	HAM10000 dataset	Decentralized	Accuracy 79%	Complexity in model training, affecting performance across images
^30^	Smart healthcare system	FL and ring signature	Kaggle dataset	Decentralized	Accuracy 91.58%	Leads to communication inefficiency for sending a large servers

Skin disease diagnostics include analyzes intensive high-resolution dermoscopic images. IoT devices with limited processing and storage capacities find it challenging to handle such data hence models that are both effective and lightweight are needed^24,25^. Additionally, the communication cost in FL frameworks exacerbates this issue, particularly when delivering large quantities of model changes in real-time from devices with constrained resources. Ethical and legal restrictions make using FL to diagnose skin conditions much more difficult^26,27^. Another issue is the lack of model interpretability, as doctors frequently want precise justifications for diagnostic judgments before they can have faith in AI systems, particularly when dealing with complex disorders like psoriasis or melanoma. The accuracy of diagnosis compromises by malicious clients who can introduce erroneous data or interfere with model updates^21–29^. The data processing techniques, robust model design, ethical adherence, and enhanced security measures are required to get over these challenges and ensure FL’s efficacy in identifying skin conditions^30–32^. FL system is used for skin disease diagnostics with an emphasis on resource utilization and data confidentiality. It eliminates the need to transfer confidential skin photos to a centralized server while working with sensitive data. This work offer four distinct models to skin disease diagnosis using DNN classifier (a) transfer learning (b) feature extraction (c) feature extraction with federated learning (d) federated transfer learning.

## Methodology skin disease diagnosis

**Fig. 1:**
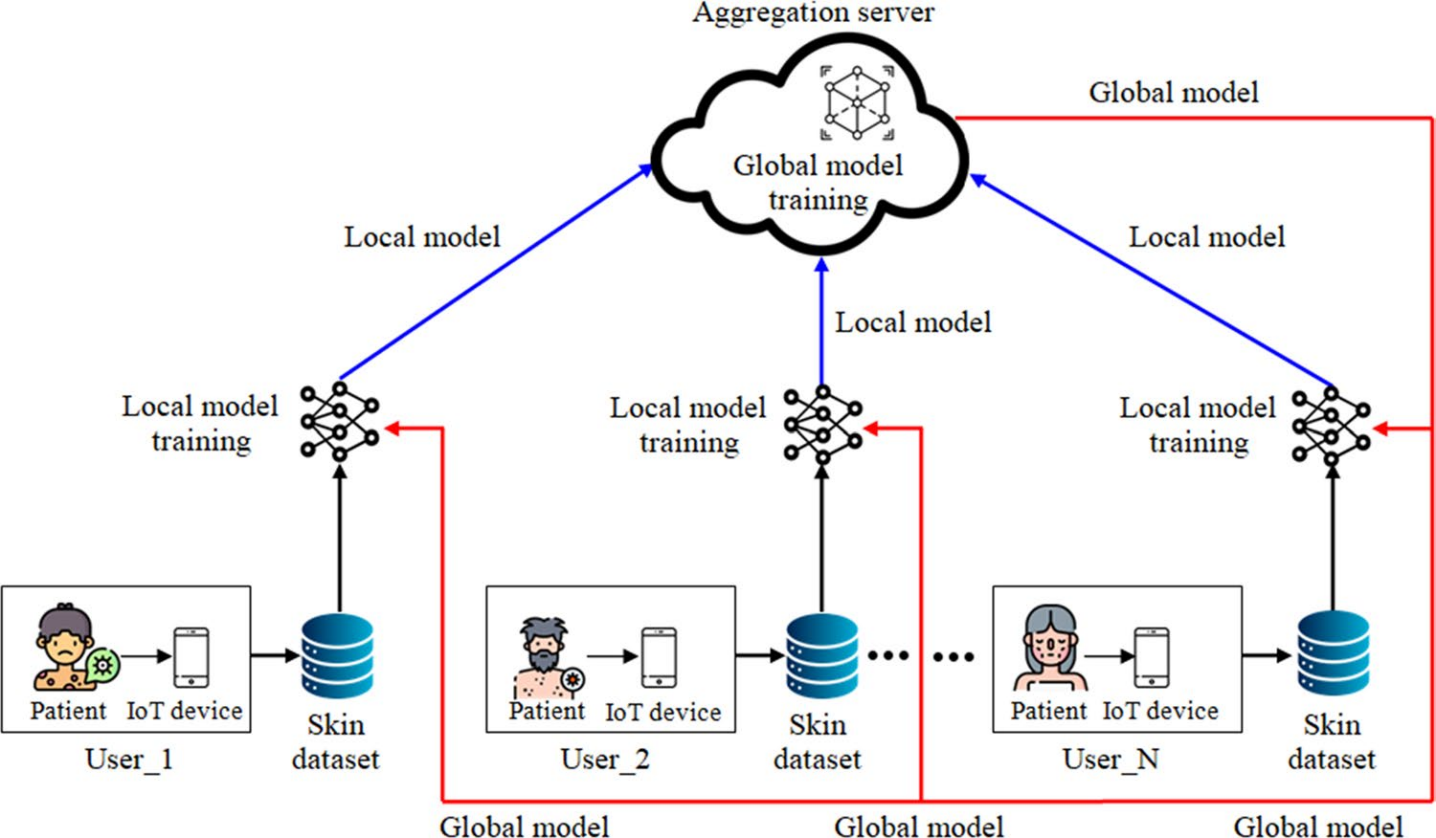
General structure of data collection from skin disease patient in FL environment.

**Fig. 2:**
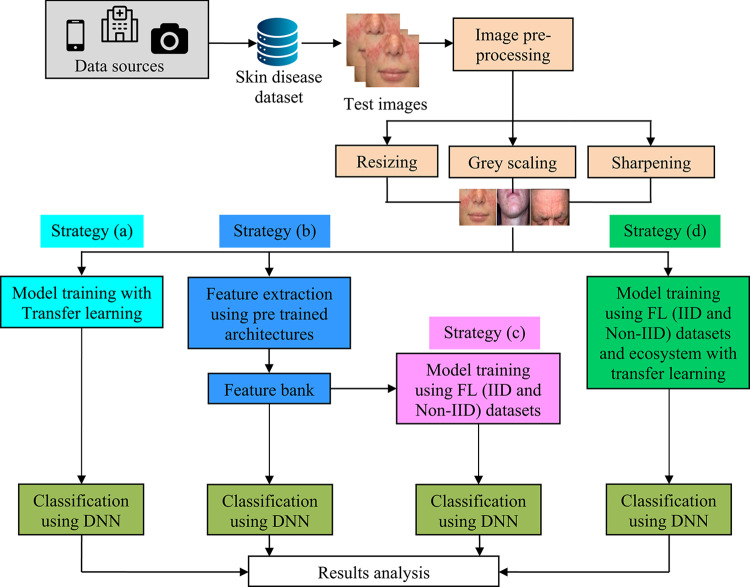
Conceptual structure of skin disease diagnosis using (**a**) transfer learning, (**b**) feature extraction, (**c**) feature extraction with federated learning, (d) federated transfer learning with DNN classifier.

This section presents a resource-efficient FL outline for the recognition and classification of skin illnesses. IoT-enabled devices at different locations collect skin disease images from patients and store them locally. The overall structure of data collection for skin disease diagnosis using FL is shown in Fig. 1. By using distributed data to enhance the accuracy of ML/DL models, it facilitates more effective diagnosis of skin conditions. Figure 2 illustrates the conceptual framework for skin disease diagnosis using four distinct strategies: federated transfer learning, feature extraction with FL, feature extraction with transfer learning, and transfer learning alone. In this framework, images of skin conditions are collected from patients across different locations and stored locally to maintain data confidentiality. Once data collection is complete, pre-processing methods—such as resizing, grayscale conversion, and sharpening—are pragmatic to reduce noise and enhance image quality. Following pre-processing, the dataset is analyzed using four methods.

1. The first method, transfer learning, employs DNN to fine-tune a pre-trained model for skin disease classification.
2. The second method combines feature extraction and transfer learning, where pre-trained models like DenseNet, VGG19, Xception, and UNet are used to extract features, which are then used for DNN-based classification.
3. The third method integrates FL and feature extraction, enabling distributed clients to collaboratively train models on both IID and non-IID datasets while ensuring strong performance and privacy.
4. The fourth method —federated transfer learning—uses FL in conjunction with transfer learning to build a global model from dispersed data while preserving patient privacy.

The proposed framework offers a secure, scalable solution to modern healthcare challenges by leveraging ML/DL methodologies in a decentralized setting.

### FL with IID and non-IID datasets

Federated learning (FL)^33^ model arrange statistics and secrecy while dealing the hitches of exercise representations above a net of detached plans. The parameters or gradients of these locally trained replicas are then collective to generate a global model. By keeping the system exercise course as adjacent to the statistics bases as likely, FL model aims to safeguard data privacy. FL model is therefore, a good optimal for submissions where secrecy is important, mainly when working with complex numbers, geographically detached evidence, or campaigns with partial possessions or erratic network connectivity. FL model has attracted a lot of consideration and research in a range of actual submissions, despite its challenges, particularly in the security domain^34^. The data-privacy-conscious industries like healthcare and finance employ FL model more frequently to overawe the confines of federal data storage. Without disclosing private patient information to outside servers, FL model enables cooperative model training in the medical field to identify illnesses^35^. FL model employs IID datasets, which have a uniform and balanced distribution of data among devices^36^, and Non-IID datasets, which have an uneven and different distribution of data between devices^37^. Real-world scenarios with inconsistent data from several sources are often reflected in non-IID databases. FL model enables resident strategies to maintain their discrete and assorted documents though attractive a universal system, even in cases when data is not disseminated evenly.

### Model training using transfer learning

In deep learning, transfer knowledge is the procedure of applying the information acquired from previously trained models to new and related situations. The key idea is to shift the focus from a large-dataset-trained model to a different-but-related goal that requires fewer labeled instances. The substantial monetary outlay needed to train intricate variables in DL models drives transfer learning. TL has becoming more and more popular in this business for good reason, and it’s easy to incorporate into real-world applications. This process retrains a trained network using just the final classification layer’s parameters by the exercise statistics from the novel mission. This study identifies skin illnesses using transfer learning models, including VGG16, Xception, EfficientNetB3, and MobileNetV2^38^. By initializing models using learned properties, transfer learning has the advantage of speeding up training and reducing computational and resource costs. CNNs are distinguished by their hierarchical representations and use of convolutional, pooling, dropout, and fully connected layers to extract features from pictures. The transmission learning model has already recognized useful traits and trends across a range of data, serving as a knowledge base. Applying the model to new work only improves the top layers; the lower layers retain all of their learnt information. Initially, transfer learning models only train on low-level structures, keeping all additional layers fixed. While training on a new dataset, transfer learning models often have their remaining layers updated or adjusted. By enabling the model to derive higher-level features pertinent to the new data distribution, altering these layers may enhance the model’s performance.When enhancing a transfer learning model, it’s important to pick your layers wisely and strike a balance between relying on prior knowledge and learning from fresh data.he empirical source distribution $\hat{Y}$ is specified as $\hat{Y} = \left\{ {\hat{Y}_{1} ,\hat{Y}_{2} , \ldots ,\hat{Y}_{K} } \right\}$, while the source circulation Y for multi-source transfer learning is definite as $Y = \{ Y_{1} ,Y_{2} , \ldots ,Y_{K} \}$, where $I_{k}$ represents the distribution of the K-th basis domain. For the set of hypothesis functions I that map P to Q, let (⋅,⋅) ∶Q × Q → R + represent the loss function. The next is the definition of the q-Discrepancy distance discY between two distributions, $I_{1}$ and $I_{2}$:

1 $${\mathrm{Disc}}_{Q} (Y_{1} ,Y_{2} ): = \mathop {\sup }\limits_{i \in I} \left| {I_{{Y_{1} }} \left( {i,F_{{Y_{1} }} } \right) - I_{{Y_{2} }} \left( {i,F_{{Y_{2} }} } \right)} \right|$$

where $I_{{x_{i} }} (i,F_{{\Gamma_{i} }} ): = e_{{p\sim x_{i} }} [(i(p),F_{{\Gamma_{i} }} (p))]$. $F_{{Y_{1} }}$ And $F_{{Y_{2} }}$ denotes the labeling meaning for the delivery $Y_{1}$ and $Y_{2}$, respectively. The empirical optimal problem of f may be clearly shown as follows, given a hypothesis class of real-valued functions f and a set of training data samples $T = \left( {t_{1} , \ldots ,t_{a} } \right)$:

2 $$\hat{R}_{T} (f) = \frac{1}{a}E\left[ {\mathop {\sup }\limits_{F \in f} \left( {\sum\limits_{h = 1}^{a} {\varepsilon_{h} } F(t_{h} )} \right)} \right]$$

where $\varepsilon = (\varepsilon_{1} , \ldots ,\varepsilon_{a} )$, ${\mathcal{E}}_{h}$ are the Rademacher random variables $X(\varepsilon_{h} = - 1) = X(\varepsilon_{h} = 1) = 0.5$. Let I be a set of theory functions i(⋅) that map the first s-time step efforts $\{ P_{1} ,P_{2} , \ldots ,P_{5} \} \in r^{{c_{n} \times 5}}$ to the s-time-step output $q_{s} \in r^{{c_{q} }}$. Using the set I and the distribution X, a new hypothesis function set $l_{I}$ is distinct as follows:

3 $$l_{I} = \left\{ {l:p \to l(i(p),F_{x} (p)),i \in I} \right\}$$

where the initial *t*-time-step inputs $p \in r^{\rho }$ are mapped to [0, 1] by the loss purpose $l(i(p),F_{X} (p)) \in l_{I}$, an $l_{R}$-Lipchitz function associated with the RNN hypothesis. The following equality holds with chance at least 1 − δ over X for every i ∈ I given a dataset of *m* samples $\hat{X} = \left( {p_{h} = q_{h} } \right)_{s - 1}^{s}$ h = 1…that is taken from the domain X:

4 $$E[l(i(p),q)] \le \frac{1}{a}\sum\limits_{h = 1}^{a} l (i(p),q) + 2\hat{R}_{T} (l_{I} ) + 3\sqrt {\frac{{\log \left( {\frac{2}{\delta }} \right)}}{2a}}$$

Particular a dataset of K divisions with $a_{g}$ examples h = 1…$a_{g}$ strained from several basis areas $Y_{g}$ for g = 1… K, the next equivalence grips with chance no less than 1 − δcompleted $Y = \{ Y_{1} ,Y_{2} , \ldots ,Y_{K} \}$ for all i ∈ I:

5 $$E[l(i(p),q)] \le \sum\limits_{g - 1}^{k} \left\{ \frac{1}{{a_{g} }}\sum\limits_{h - 1}^{{a_{g} }} l (i(p),q) + 2\hat{R}_{{T_{g} }} (l_{1} ) + 3\sqrt {\frac{{\log \left( {\frac{2K}{\delta }} \right)}}{{2a_{g} }}} \right\}$$

The next variation grips for $Y_{g}$ with chance no less than 1 − δ∕K using δ∕K in its place of δ for g = 1, … K.

6 $$E[l(i(p),q)] \le \frac{1}{{a_{g} }}\sum\limits_{h = 1}^{{a_{x} }} l (i(p),q) + 2\hat{R}_{{T_{x} }} (l_{I} ) + 3\sqrt {\frac{{\log \left( {\frac{2K}{\delta }} \right)}}{{2a_{g} }}}$$

samples $\hat{Q} = \left( {p_{h}^{g} \cdot q_{h}^{g} } \right)_{s - 1}^{S}$,h = 1,…, $a_{g}$, from the basis domain $Y_{g}$ for g = 1, …, K, and set of data samples $\hat{X} = \left( {p_{h} = q_{h} } \right)_{s - 1}^{s}$,h = 1,…,a, drawn from the aim area X. The triangle inequality and the definition were used to compute the Q-discrepancy distance. The following equation can further constrain the major component in the next line with a probability of at least 1 − δ over X, according to the goal function.

7 $$Disc_{Q} (X,\hat{X}) = |l_{{\hat{X}}} (i,F_{X} ) - l_{X} (i,F_{X} )| \le 2\hat{R}_{T} (l_{I} ) + 3\sqrt {\frac{{\log \left( {\frac{2K}{\delta }} \right)}}{2a}}$$

Additionally, the bound for $Disc_{Q} (Y,\hat{Y})$

8 $$Disc_{Q} (Y,\hat{Y}) = |l_{{\hat{Y}}} (i,F_{Y} ) - l_{Y} (i,F_{Y} )| \le \sum\limits_{h = 1}^{k} {\left[ {2\hat{R}_{{T_{h} }} (l_{I} ) + 3\sqrt {\frac{{\log \left( {\frac{2K}{\delta }} \right)}}{{2a_{h} }}} } \right]}$$

Let me be a private of the theoretical purpose i(⋅) that translates the concept of the RNN to the output of the s-th step. The following difference grips with a chance of at least 1 − δ: Set a dataset of K subsections with samples $\hat{Q} = \left( {p_{h}^{g} \cdot q_{h}^{g} } \right)_{s - 1}^{S}$ h = 1,…, to from the base domain $Y_{g}$ for g = 1,…, K, and a set of data samples $\hat{X} = \left( {p_{h} = q_{h} } \right)_{s - 1}^{s}$, h = 1,…,a, pulled from the objective area X.

9 $$l_{X} (i,F_{X} ) - l_{{\hat{Y}}} (i,F_{Y} ) = + \mathop {\sup }\limits_{i \in I} \left|e_{{p\sim \hat{X}}} [l(i(p),F_{{\hat{x}}} (p))] - \frac{{\sum\limits_{h - 1}^{K} {a_{h} } e_{{p\sim \hat{Y}_{h} }} [l(i(p),F_{{\hat{Y}_{h} }} (p))]}}{{\sum\limits_{h - 1}^{K} {a_{h} } }}\right|$$

The triangle inequality condition and the discrepancy distance concept may be used to get the following inequality.

10 $$\begin{gathered} l_{X} (i,F_{X} ) = l_{X} (i,F_{X} ) - l_{Y} (i,F_{Y} ) + l_{Y} (i,F_{Y} ) - l_{{\hat{Y}}} (i,F_{Y} ) + l_{{\hat{Y}}} (i,F_{Y} ) \hfill \\ \le Disc_{Q} (X,Y) + Disc_{Q} (Y,\hat{Y}) + l_{{\hat{Y}}} (i,F_{Y} ) \hfill \\ \le l_{{\hat{Y}}} (i,F_{Y} ) + 2Disc_{Q} (Y,\hat{Y}) + Disc_{Q} (\hat{X},\hat{Y}) + Disc_{Q} (X,\hat{X}) \hfill \\ \end{gathered}$$

Q-discrepancy distance and characteristics of empirical source distribution $\hat{Y} = \left\{ {\hat{Y}_{1} ,\hat{Y}_{2} , \ldots ,\hat{Y}_{K} } \right\}$ defined as follows.

11 $$Disc_{{\mathcal{Q}}} (\hat{X},\hat{Y}) = \mathop {\sup }\limits_{i \in I} \left|e_{{p\sim \hat{X}}} [l(i(p),F_{{\hat{i}}} (p))] - \frac{{\sum\nolimits_{h = 1}^{K} {a_{h} } e_{{p\sim \hat{Y}_{h} }} [l(i(p),F_{{\hat{Y}_{h} }} (p))]}}{{\sum\nolimits_{h = 1}^{K} {a_{h} } }}\right|$$

Lastly, the inequality that follows may be obtained using

12 $$l_{X} (i,F_{X} ) = \mathop {\sup }\limits_{i\epsilon I} \left| {e_{{p\sim \hat{X}}} [l(i(p),F_{X} (p))] - \frac{{\sum\nolimits_{h = 1}^{k} {a_{h} } e_{{p\sim \hat{Y}_{h} }} [l(i(p),F_{{\hat{Y}_{h} }} (p))]}}{{\sum\nolimits_{h = 1}^{k} {a_{h} } }} + 6\sum\nolimits_{h = 1}^{k} {\sqrt {\frac{{\log \left( {\frac{2K + 2}{\delta }} \right)}}{{2a_{h} }}} } } \right|$$

The empirical error of the function i as evaluated on the experimental multi-source area $\hat{Y}$ is represented. $\hat{X}$ And $\hat{Y}$ is Q-discrepancy distance is the second term. The function set I on the empirical basis domain $\hat{Y}$ and the empirical goal domain $\hat{X}$ has a Rademacher difficulty term that is the third and fourth terms, respectively. The final two elements show the probability terms, which are based on the assurance level δ and the quantity of data samples.

### Feature extraction using pre-trained architectures

A key component of DL models that enables effective use of a pre-conditioned neuronal system’s abilities is feature extraction. Among the several layers in these networks that are especially built to extract essential characteristics for tasks like object identification and localization are convolutional and pooling layers. To might change the learning rate, add layers, and variation the sum of neurons in every stratum, and so on to advance our systems. These methods provide significant time and computing resource savings. Pre-trained replicas that have been trained on huge datasets are effective feature extractors. System performance can be improved by selecting the appropriate feature extractor. DenseNet, VGG19, Xception, and UNet were among the pre-trained models^39^ used which are used to extract the properties of the second-to-last layers. The resulting attributes are then used to classify skin illnesses in FL with IID and Non-IID databases. By allowing remote devices to work together by sharing these derived features for model training, they excel at extracting meaningful patterns from high-dimensional image data, such as lesions’ shape, colour, texture, and edge details, which are critical for diagnosing skin conditions.

### Classification using dense neural network (DNN)

Dense neural network (DNN) is highly effective in performing complex classification tasks and learning intricate data representations^40^. A DNN can learn hierarchical features from input data because it has several completely linked layers, with each neuron in one layer connected to every other neuron in the layer above. In this context, DNNs are particularly advantageous. As the input features propagate deeper into the network, higher layers extract more abstract and disease-specific patterns, enabling accurate differentiation between various skin conditions. To simulate intricate relationships in the data, each layer of a DNN applies a weighted sum and then a non-linear activation function.The model is trained using supervised learning with labeled datasets, optimizing weights via backpropagation and gradient descent to minimize classification error. The architecture’s ability to learn deep, abstract features makes it well-suited for skin disease diagnosis, where subtle variations in texture, color, and lesion shape can significantly affect classification accuracy. By leveraging the dense connectivity of DNNs, the system achieves robust performance in automated dermatological analysis. The architecture consists of three main layers: the input, hidden, and the output layer. The input layer is the first point of contact for the model and receives raw data, which, in the context of skin disease classification, typically includes a feature vector derived from skin images. The hidden layers form the core computational engine of the DNN, where each layer applies a weighted sum followed by a non-linear ReLU activation function. The first hidden layer focuses on detecting low-level features like edges, spots, and gradients, which serve as fundamental building blocks in image recognition. Subsequent intermediate layers learn to combine these low-level cues into more complex structures such as textures, shapes, and boundary patterns that are often characteristic of specific skin conditions. The model’s depth and width significantly influence its ability to generalize across diverse cases, although deeper networks may require larger datasets and robust regularization to mitigate overfitting. The output layer gives the classification result, typically using a softmax activation function to generate chances for each class.The input and output feature maps of a precise layer can be characterized as $P \in r^{I \times Z \times H}$ and $Q \in r^{I \times Z \times O}$, where I, Z, H, and O represent the height, width, and number of channels, individually. The convolutional filters are embodied as $D \in r^{I \times O}$. In group involvedness, the feature maps P, Q , and the filters D are separated into G distinct groups. Group convolution is characterized in the calculations below. Here ⊗ characterizes 2D convolution.

13 $$Q^{j} = P^{j} \otimes D^{j}$$

14 $$Q = Q^{1} \cup Q^{2} \cup \ldots \cup Q^{j} \ldots \cup Q^{l}$$

The depth-dependent convolution used in the DNN module allows for the extraction of localized features while preserving the spatial scale of the data. The subsequent point-wise dense vector further improves the replica’saptitude to acquiremultifaceted representations by combining features from different channels, allowing for richer information encoding. Depth convolution and point convolution is describes as follows.

15 $$dc(Z,q)_{(h,g)} = \sum\nolimits_{K,L}^{k,l} {Z_{(K,L)} } \times q_{(h + K,g + L)}$$

16 $$pc(Z,q)_{(h,g)} = \sum\nolimits_{a}^{A} {Z_{a} } q_{a}$$

Here Z denotes the difficulty kernel, q denotes the contributionarticle map, h and g are the dimensions of the input feature map, K and L are the dimensions of the output feature map, and m denotes the number of channels. Triple attention (TA) improves the replica’scapability to recognize and discriminate different characteristics. Each branch is used to analyze the input tensor (χ ∈ RC × I × Z) in different ways, which improves the model’s complex shapes. In each branch, the input tensor undergoes rotation, followed by W-union and convolution operations, which help extract dimensional correlations between height and channel dimensions. The W-pool function is given by the following relation.

17 $$W = {\mathrm{Pool}}\;(p) = [\max {\mathrm{pool}}_{{0_{c} }} (p),\;{\mathrm{avg}}\;{\mathrm{pool}}_{{0_{c} }} (p)]$$

By capturing key interactions between features at dissimilarbalances and locations, TA improves the replica’s ability to identify subtle patterns essential for accurate classification. The final refined feature map is generated by averaging the refined tensors generated by each branch.

18 $$q = \frac{1}{3}\left( {\overline{{\hat{\chi }_{1} \sigma \left( {\psi_{1} \left( {\hat{\chi }_{1}^{*} } \right)} \right)}} + \overline{{\left( {\hat{\chi }_{2} \sigma \left( {\psi_{2} \left( {\hat{\chi }_{2}^{*} } \right)} \right)} \right)}} + \left( {P\sigma \left( {\psi_{3} \left( {\hat{\chi }_{3} } \right)} \right)} \right)} \right)$$

whereσ represents the sigmoid function of each objective while $\psi_{1}$,$\psi_{2}$ and $\psi_{3}$ denotes the average two dimensional convolutional layers definite by kernel size K in the three twigs of triplet courtesy.

19 $$q = \frac{1}{3}(\overline{{\hat{\chi }_{1} \omega_{1} }} + \overline{{\hat{\chi }_{2} \omega_{2} }} + \chi \omega_{3} ) = \frac{1}{3}(\overline{{q_{1} }} + \overline{{q_{2} }} + \overline{{q_{3} }} )$$

where $\omega_{1}$, $\omega_{2}$, and $\omega_{3}$ represents the three-dimensional attention weights $q_{1}$ and $q_{2}$ which ensures that TA effectively captures spatial and channel dependencies. The working process of skin disease diagnosis using DNN is summarized in Algorithm 1.

**Figure:**
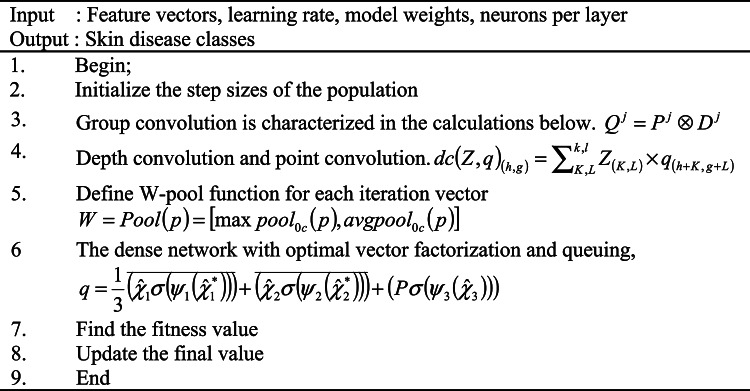
**Algorithm 1**: Skin disease diagnosis using DNN

## Results analysis

This segment presents the results and comparative examination of the models used to identify skin illnesses. Parameters such as accuracy, precision, recall, and loss are used to measure how effectively the models detect the specified skin diseases. The proposed FL model implemented on the Google Colab platform using Python, with model training and testing conducted on Colab cloud GPU server. Given the size of the HAM10000 dataset and the iterative communication between local and global models in FL, model training requires substantial computational time, which CPU cannot efficiently handle. For system-level validation, experiments were also executed on a local system configuration comprising an NVIDIA GTX 1650 graphics card with 4 GB dedicated memory, 16 GB RAM, and an Intel Core i5 processor. In order to adjust volume of time, the model is often built and executed on a GPU. An existing FL sample available on Kaggle.com was adapted and modified to design the FL framework used in this study. The FedAvg method is used to average all of the local networks in order to aggregate them into a global network at the FL server.HAM10000 "Human against Machine with 10,000 training images^41^," a publicly accessible resource housed in the ISIC repository, served as the dataset used. Regarding hyperparameter tuning, all models trained using hyperparameters optimized through empirical tuning and grid search experiments. Specifically, the learning rate, batch size, and number of epochs were systematically varied for each model to achieve optimal performance on the validation dataset. During tuning, the number of epochs was varied from 0 to 150, and the best-performing configuration was selected based on accuracy and convergence behavior. For most models, a learning rate of 0.001, batch size of 32, and 100 epochs were found to provide the most effective balance between training time and model performance.

**Fig. 3:**
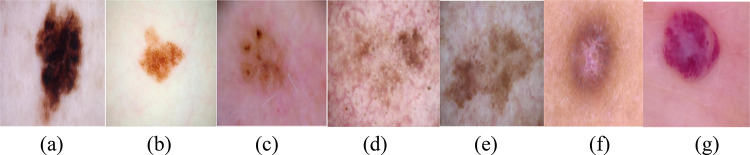
Skin images from dataset (**a**) MEL, (**b**) MV, (**c**) BCC, (**d**) AK, (**e**) BKL, (**f**) DF, (**g**) VL.

**Fig. 4:**
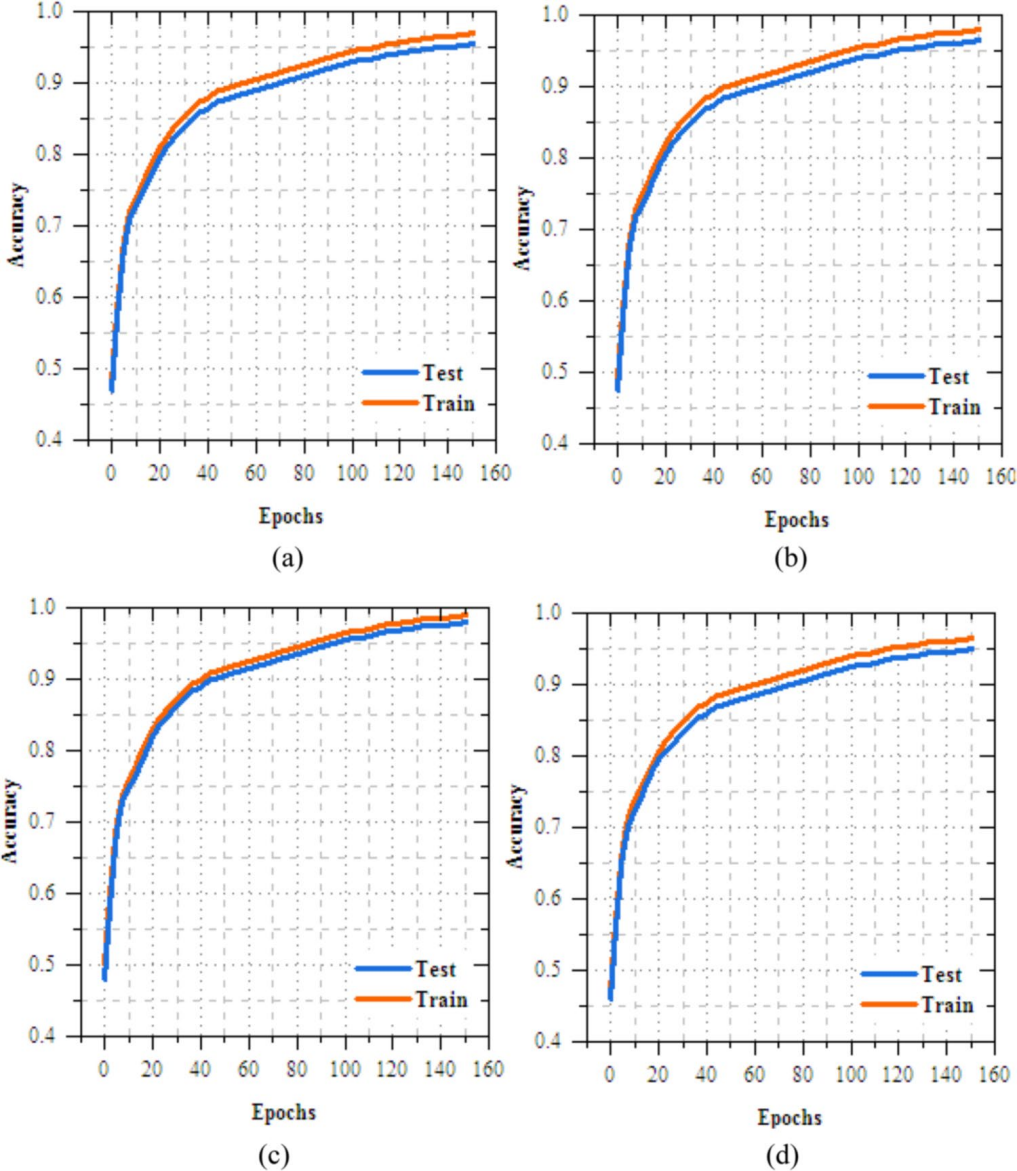
Training and testing accuracy of transfer learning models for skin disease diagnosis (**a**) VGG16, (**b**) Xception, (**c**) EfficientNetB3 and (**d**) MobileNetV2.

The dataset includes 11,253 dermatoscopeimages that show seven dissimilar kinds of skin infections (Fig. 3): vascular lesions 412, benign keratosis-like lesions 1058, basal cell carcinoma 358, actinic keratosis 6858, melanocytic nevi 635, melanoma 847, and Dermatofibroma 1085 (Table 2). The training and testing groups were randomly selected from the dataset. Ten percent of the dataset is used for testing, while ninety percent is used for training. To prevent overfitting during training, a validation process was also included. FL used both IID database, where records is disseminated consistently and identically among devices, and Non-IID database, where data spreading is uneven and differs amongst devices, as shown in Fig. 4 for 2 distinct clients (N = 2). Non-IID databases often depict real-world situations with conflicting information from several sources.Every client uses its own local dataset to train on its own network. The server receives all of the local networks and combines them into a global network once the local networks have finished training. The neural network is subsequently distributed back to the customers. The clients then train their local network once more using their local dataset, utilizing the global network as a fresh starting point. The cycle is repeated 100 times once the client’s local network has been upgraded. The model presented in this work assumes that there are no problems with the communication between the clients and the server. In practice, a local network to global network transfer can be costly and erratic, which increases the likelihood of mistakes.

**Table 2:** Number of images in dataset.

Skin disease name	Number of images
Melanocytic nevi (MV)	635
Melanoma (MEL)	847
Benign keratosis-like lesions (BKL)	1058
Basal cell carcinoma (BCC)	358
Actinic keratoses (AK)	6858
Vascular lesions (VL)	412
Dermatofibroma (DF)	1085

### Results analysis of transfer learning models on skin disease diagnosis

**Fig. 5:**
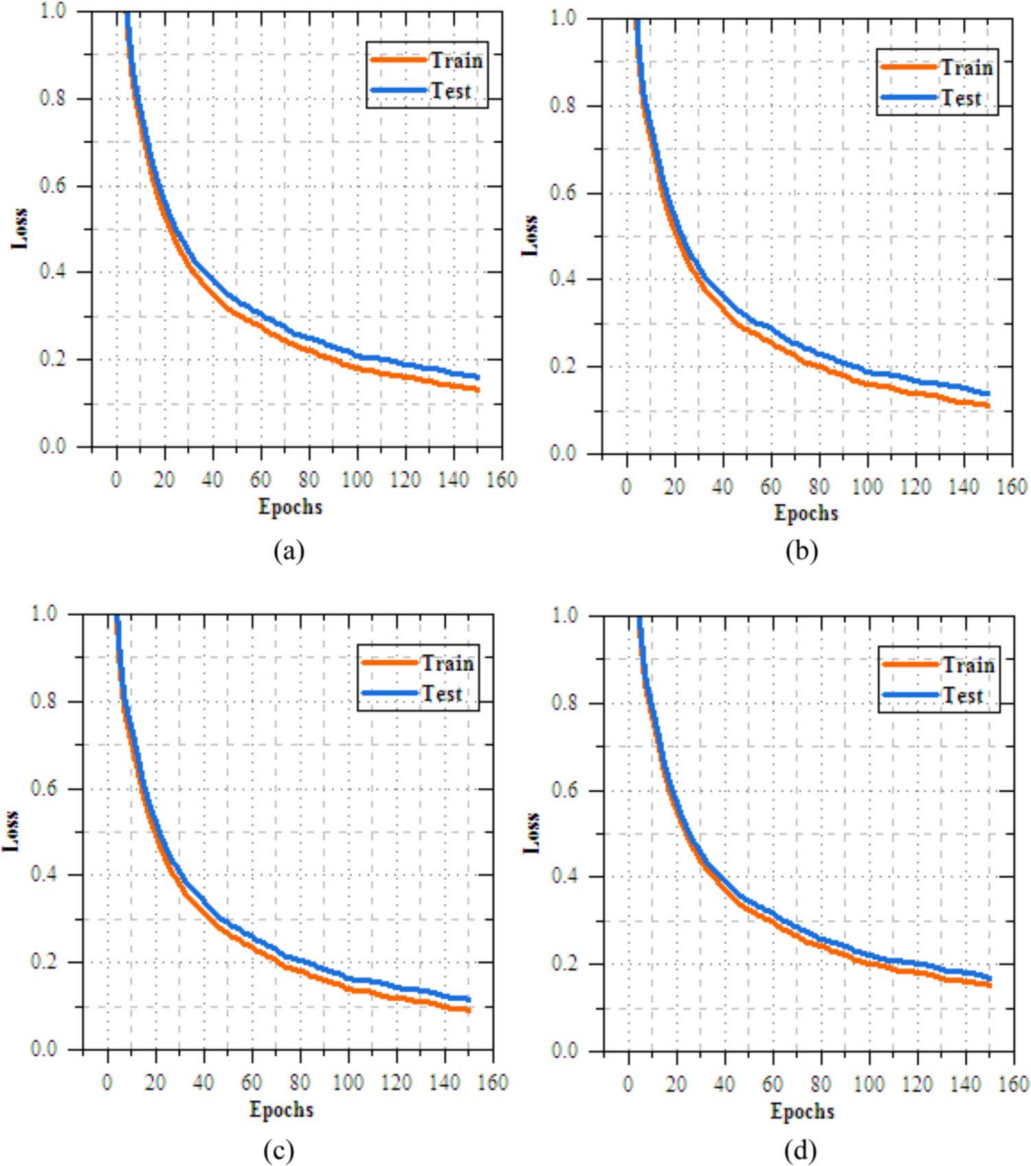
Training and testing accuracy of transfer learning models for skin disease diagnosis (**a**) VGG16, (**b**) Xception, (**c**) EfficientNetB3 and (**c**) MobileNetV2.

This section provides a detailed analysis of transmissionknowledgereplicas for casing illness diagnosis. Figure 4 shows the results analysis of training and testing accuracy for VGG16, Xception, EfficientNetB3, and MobileNetV2 reveals that MobileNetV2 performs best, with a high training accuracy of 90.352% and testing accuracy of 98.374%. Xception follows closely, achieving 87.798% in training and 97.985% in testing, while EfficientNetB3 reaches 89.857% in training and 97.968% in testing. VGG16, despite some fluctuations, achieves a strong testing accuracy of 95.858%, but with slower convergence. MobileNetV2 outperforms the others, offering the best accuracy and generalization for skin disease classification. Fig. 5 demonstrations the loss results of the transfer learning models during training and exciting over 10 epochs. Among the models, MobileNetV2 shows the best performance, with training loss reduced from 0.258 to 0.175 and testing loss from 0.199 to 0.116, reflecting its strong learning and generalization capabilities. EfficientNetB3 follows closely, with consistent reductionin training and testing loss to 0.235 and 0.076, respectively. Xception demonstrates moderate progress, ending with testing loss of 0.077, while VGG16 shows slower improvement, with a final testing loss of 0.966. MobileNetV2 and EfficientNetB3 is the most efficient models, with MobileNetV2 achieving the lowest losses, making suitable for the skin disease diagnosis task.

Table 3 delivers a relativeexamination of transfer knowledge models with DNN classification for skin disease detection. MobileNetV2 achieves the highest testing accuracy at 98.064%, shown 3.45% increase over EfficientNetB3 and 3.64% improvement compared to Xception. To address this, regularization techniques were applied, including dropout (rate 0.5), early stopping (patience = 10), and batch normalization. The results in Table 4 present the class-wise accuracy of transfer learning models with DNN, including VGG16 + DNN, Xception + DNN, EfficientNetB3 + DNN, and MobileNetV2 + DNN, across 10 folds of K-fold cross-validation for skin disease diagnosis. Table 5 presents a comparative analysis of resource metrics for transfer learning models utilized in skin disease detection, evaluating GPU memory usage, GPU process usage, CPU process usage, and virtual memory consumption.

**Table 3:** Results comparison of transfer learning models for skin disease diagnosis.

Transfer learning models	Accuracy (%)		Loss	
	Training	Testing	Training	Testing
VGG16	94.805	84.281	0.768	0.785
Xception	96.695	87.039	0.625	0.655
EfficientNetB3	97.611	88.09	0.648	0.663
MobileNetV2	98.064	91.53	0.498	0.502

**Table 4:** Class-wise accuracy of proposed transfer learning with DNN models for skin disease diagnosisover k-fold cross validation.

Class	Model	K-fold cross validation									
		1	2	3	4	5	6	7	8	9	10
MV	VGG16 + DNN	94.811	94.796	94.802	94.815	94.789	94.808	94.803	94.797	94.814	94.791
	Xception + DNN	96.701	96.688	96.695	96.709	96.682	96.703	96.690	96.697	96.705	96.684
	EfficientNetB3 + DNN	97.616	97.605	97.612	97.620	97.598	97.614	97.607	97.619	97.601	97.615
	MobileNetV2 + DNN	98.069	98.057	98.064	98.073	98.050	98.068	98.061	98.070	98.055	98.066
MEL	VGG16 + DNN	94.799	94.813	94.805	94.790	94.816	94.801	94.807	94.794	94.812	94.798
	Xception + DNN	96.689	96.702	96.695	96.680	96.710	96.691	96.698	96.685	96.706	96.693
	EfficientNetB3 + DNN	97.604	97.617	97.611	97.597	97.621	97.608	97.613	97.602	97.619	97.605
	MobileNetV2 + DNN	98.058	98.071	98.064	98.049	98.075	98.060	98.067	98.053	98.072	98.059
BKL	VGG16 + DNN	94.807	94.792	94.815	94.803	94.798	94.810	94.795	94.814	94.800	94.806
	Xception + DNN	96.697	96.683	96.709	96.701	96.688	96.705	96.691	96.708	96.694	96.702
	EfficientNetB3 + DNN	97.613	97.599	97.620	97.615	97.604	97.618	97.601	97.616	97.607	97.614
	MobileNetV2 + DNN	98.065	98.051	98.074	98.068	98.057	98.071	98.060	98.073	98.055	98.067
BCC	VGG16 + DNN	94.804	94.811	94.796	94.809	94.802	94.815	94.797	94.813	94.800	94.808
	Xception + DNN	96.694	96.707	96.681	96.703	96.696	96.710	96.685	96.708	96.692	96.705
	EfficientNetB3 + DNN	97.610	97.623	97.597	97.616	97.612	97.625	97.603	97.619	97.608	97.614
	MobileNetV2 + DNN	98.062	98.076	98.048	98.070	98.065	98.078	98.054	98.072	98.060	98.067
AK	VGG16 + DNN	94.812	94.795	94.809	94.801	94.814	94.797	94.806	94.803	94.811	94.799
	Xception + DNN	96.706	96.684	96.702	96.694	96.709	96.687	96.705	96.698	96.712	96.690
	EfficientNetB3 + DNN	97.618	97.596	97.614	97.607	97.621	97.600	97.616	97.609	97.624	97.603
	MobileNetV2 + DNN	98.072	98.049	98.067	98.060	98.075	98.053	98.070	98.063	98.077	98.056
VL	VGG16 + DNN	94.798	94.815	94.801	94.807	94.794	94.812	94.805	94.809	94.796	94.813
	Xception + DNN	96.682	96.708	96.693	96.699	96.686	96.711	96.695	96.702	96.684	96.707
	EfficientNetB3 + DNN	97.600	97.622	97.605	97.611	97.597	97.624	97.608	97.615	97.602	97.620
	MobileNetV2 + DNN	98.053	98.076	98.058	98.065	98.050	98.078	98.061	98.068	98.055	98.073
DF	VGG16 + DNN	94.805	94.790	94.813	94.797	94.811	94.803	94.808	94.795	94.814	94.801
	Xception + DNN	96.695	96.680	96.709	96.692	96.706	96.698	96.704	96.687	96.711	96.693
	EfficientNetB3 + DNN	97.611	97.597	97.624	97.603	97.618	97.609	97.615	97.601	97.622	97.607
	MobileNetV2 + DNN	98.064	98.050	98.077	98.056	98.071	98.062	98.068	98.054	98.075	98.060

**Table 5:** Resource metrics of transfer learning models for skin disease detection.

	Minimum	Maximum	Average	Minimum	Maximum	Average
Model	GPU Memory Used (%)			GPU Process Used (%)		
VGG16	73.250	77.850	75.550	66.350	70.850	68.600
Xception	72.950	77.350	75.150	65.880	71.250	68.565
EfficientNetB3	73.580	78.120	75.850	66.750	70.950	68.850
MobileNetV2	73.850	78.550	76.200	67.150	71.450	69.300
	CPU Process Used (%)			Virtual Memory Used (%)		
VGG16	10.250	11.950	11.100	79.120	82.580	80.850
Xception	9.850	11.750	10.800	78.580	82.250	80.415
EfficientNetB3	10.650	11.880	11.265	79.450	83.150	81.300
MobileNetV2	10.850	12.050	11.450	80.250	83.480	81.865

### Results analysis of feature extraction models on skin disease diagnosis

**Fig. 6:**
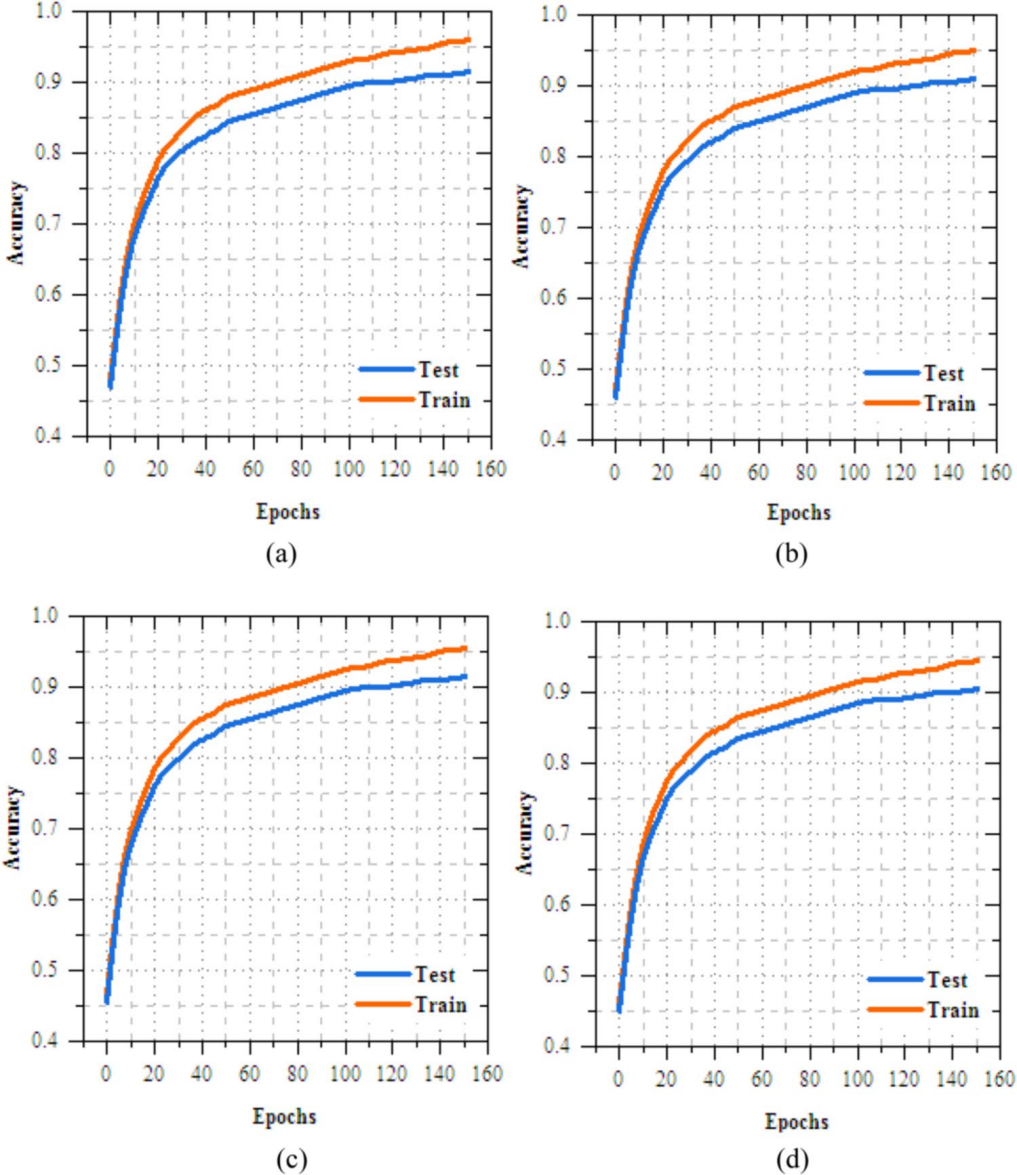
Training and testing accuracy of feature extortion models for skin disease diagnosis (**a**) DenseNet, (**b**) VGG19, (**c**) Xception and (**d**) UNet.

**Fig. 7:**
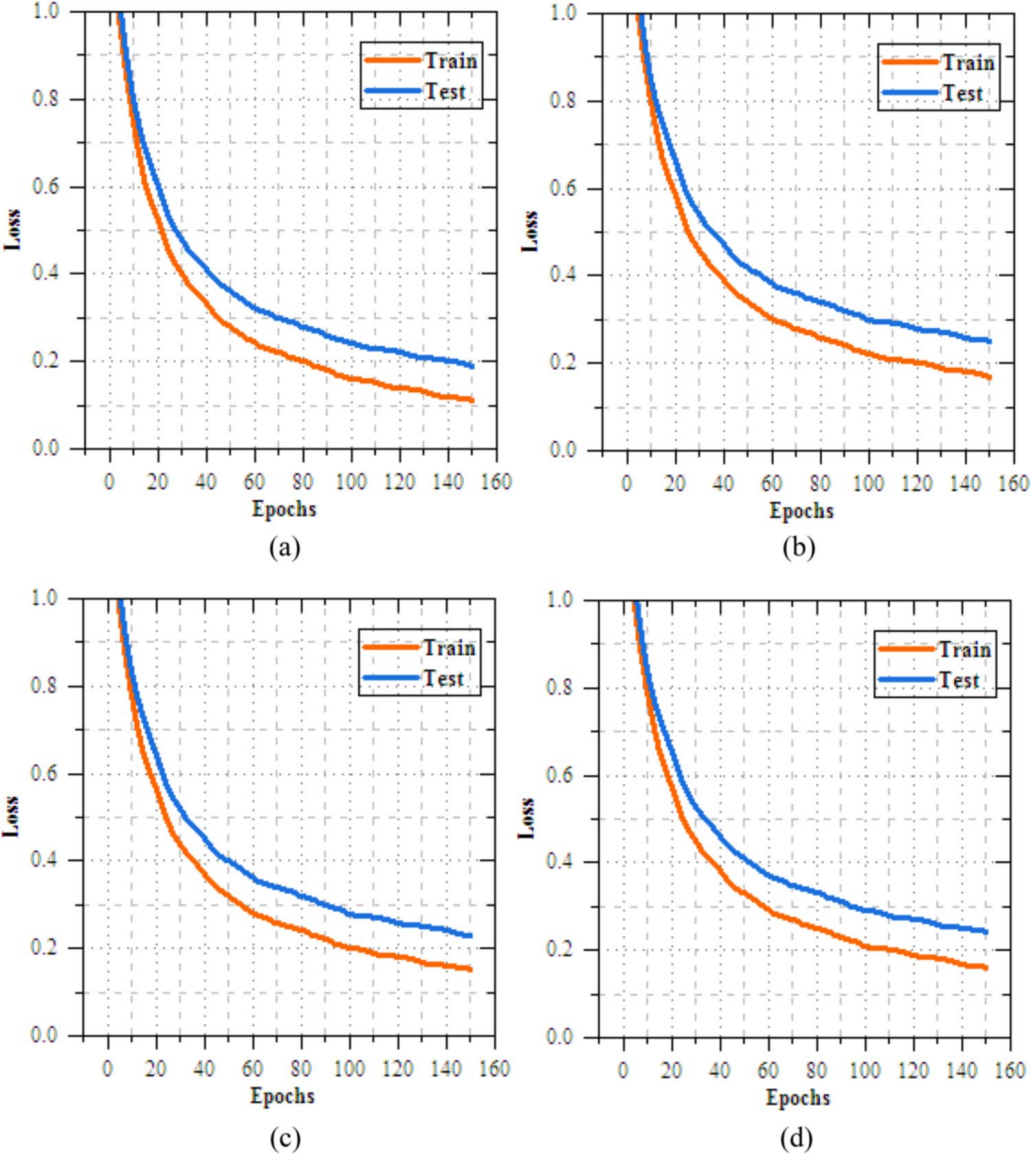
Training and testing loss of feature extortion models for skin disease diagnosis (**a**) DenseNet, (**b**) VGG19, (**c**) Xception and (**d**) UNet.

Figure 6 compares the accuracy of DenseNet, VGG19, Xception, and UNet during training and testing. UNet leads with the highest exercise accuracy of 84.537% and challengingexactness of 90.894%, presentation strong generalization. VGG19 improves steadily, reaching 82.845% in training and 89.202% in testing, while DenseNet trails with peak training accuracy of 82.365% and testing accuracy of 88.722%. Figure 7 shows that UNet also achieves the lowest loss during both phases, reducing training loss to 0.406 and testing loss to 0.321 by epoch 50. Table 6 presents the results of feature extraction models with a DNN classifier for skin disease discovery and organization, highlighting notable differences in both accuracy and loss metrics. In terms of loss values, UNet achieves the lowest testing loss at 0.112, followed by Xception at 0.124, VGG19 at 0.158, and DenseNet at 0.138. UNet reduces testing loss by 0.026 compared to DenseNet, 0.046 compared to VGG19, and 0.012 compared to Xception. For training loss, UNet again records the lowest value of 0.087, while Xception follows with 0.098, VGG19 at 0.145, and DenseNet at 0.125. UNet reduces training loss by 0.038 over DenseNet, 0.058 over VGG19, and 0.011 over Xception.

**Table 6:** Results comparison of feature extraction models for skin disease diagnosis.

Feature extraction model	Accuracy (%)		Loss	
	Training	Testing	Training	Testing
DenseNet	87.85	81.494	0.625	0.638
VGG19	88.713	82.28	0.645	0.658
Xception	89.511	83.067	0.598	0.624
UNet	**90.338**	**83.854**	0.587	0.612

The results in Table 7 shows the class-wise accuracy of feature extraction models with DNN (DenseNet + DNN, VGG19 + DNN, Xception + DNN, UNet + DNN) across 10 folds of K-fold cross-validation for skin disease diagnosis. UNet + DNN achieve the highest average accuracy, ranging from 90.31% to 90.37% across all classes (MV, MEL, BKL, BCC, AK, VL, and DF), indicating superior performance. Table 8 presents the resource utilization metrics for feature extraction models integrated with DNN classification frameworks for skin disease detection and classification, focusing on GPU memory, GPU process, CPU process, and virtual memory usage. UNet demonstrates higher GPU memory, GPU process, CPU process, and virtual memory usage, making it more computationally demanding but potentially suited for scenarios requiring higher processing capabilities.

**Table 7:** Class-wise accuracy of proposed feature extraction with DNN models for skin disease diagnosis over k-fold cross validation.

Class	Model	K-fold cross validation									
		1	2	3	4	5	6	7	8	9	10
MV	DenseNet + DNN	87.832	87.865	87.841	87.879	87.823	87.856	87.848	87.870	87.834	87.862
	VGG19 + DNN	88.695	88.728	88.704	88.742	88.687	88.719	88.711	88.735	88.698	88.726
	Xception + DNN	89.493	89.526	89.502	89.540	89.485	89.517	89.509	89.533	89.496	89.524
	UNet + DNN	90.320	90.353	90.329	90.367	90.312	90.344	90.336	90.360	90.323	90.351
MEL	DenseNet + DNN	87.859	87.836	87.872	87.844	87.867	87.829	87.855	87.841	87.863	87.837
	VGG19 + DNN	88.722	88.699	88.737	88.709	88.731	88.694	88.720	88.706	88.729	88.701
	Xception + DNN	89.520	89.497	89.535	89.507	89.529	89.492	89.518	89.504	89.527	89.499
	UNet + DNN	90.347	90.324	90.362	90.334	90.356	90.319	90.345	90.331	90.354	90.326
BKL	DenseNet + DNN	87.840	87.873	87.849	87.831	87.864	87.846	87.868	87.854	87.827	87.851
	VGG19 + DNN	88.703	88.736	88.712	88.694	88.727	88.709	88.732	88.718	88.691	88.715
	Xception + DNN	89.501	89.534	89.510	89.492	89.525	89.507	89.530	89.516	89.489	89.513
	UNet + DNN	90.328	90.361	90.337	90.319	90.352	90.334	90.357	90.343	90.316	90.340
BCC	DenseNet + DNN	87.866	87.843	87.825	87.858	87.880	87.852	87.834	87.869	87.847	87.829
	VGG19 + DNN	88.729	88.706	88.688	88.721	88.743	88.715	88.697	88.732	88.710	88.692
	Xception + DNN	89.527	89.504	89.486	89.519	89.541	89.513	89.495	89.530	89.508	89.490
	UNet + DNN	90.354	90.331	90.313	90.346	90.368	90.340	90.322	90.357	90.335	90.317
AK	DenseNet + DNN	87.847	87.870	87.842	87.864	87.836	87.859	87.841	87.875	87.853	87.828
	VGG19 + DNN	88.710	88.733	88.705	88.727	88.699	88.722	88.704	88.738	88.716	88.691
	Xception + DNN	89.508	89.531	89.503	89.525	89.497	89.520	89.502	89.536	89.514	89.489
	UNet + DNN	90.335	90.358	90.330	90.352	90.324	90.347	90.329	90.363	90.341	90.316
VL	DenseNet + DNN	87.854	87.831	87.867	87.839	87.862	87.844	87.876	87.848	87.830	87.855
	VGG19 + DNN	88.717	88.694	88.730	88.702	88.725	88.707	88.739	88.711	88.693	88.718
	Xception + DNN	89.515	89.492	89.528	89.500	89.523	89.505	89.537	89.509	89.491	89.516
	UNet + DNN	90.342	90.319	90.355	90.327	90.350	90.332	90.364	90.336	90.318	90.343
DF	DenseNet + DNN	87.861	87.838	87.874	87.846	87.868	87.850	87.832	87.857	87.879	87.841
	VGG19 + DNN	88.724	88.701	88.737	88.709	88.731	88.713	88.695	88.720	88.742	88.706
	Xception + DNN	89.522	89.499	89.535	89.507	89.529	89.511	89.493	89.518	89.540	89.504
	UNet + DNN	90.349	90.326	90.362	90.334	90.356	90.338	90.320	90.345	90.367	90.331

**Table 8:** Resource metrics results of feature extraction models with DNN classification for skin disease detection and classification.

	Minimum	Maximum	Average	Minimum	Maximum	Average
Model	GPU memory used (%)			GPU process used (%)		
DenseNet	66.250	71.450	68.850	58.360	63.580	60.970
VGG19	65.850	70.680	68.265	58.120	62.750	60.435
Xception	66.480	71.120	68.800	59.450	63.250	61.350
UNet	67.120	71.880	69.500	60.120	64.120	62.120
	CPU process used (%)			Virtual memory used (%)		
DenseNet	7.550	9.250	8.400	75.360	78.450	76.905
VGG19	7.250	8.950	8.100	74.650	77.850	76.250
Xception	7.650	9.350	8.500	75.580	78.950	77.265
UNet	7.850	9.580	8.715	76.120	79.150	77.635

### Results analysis of federated transfer learning models on skin disease diagnosis

**Fig.8:**
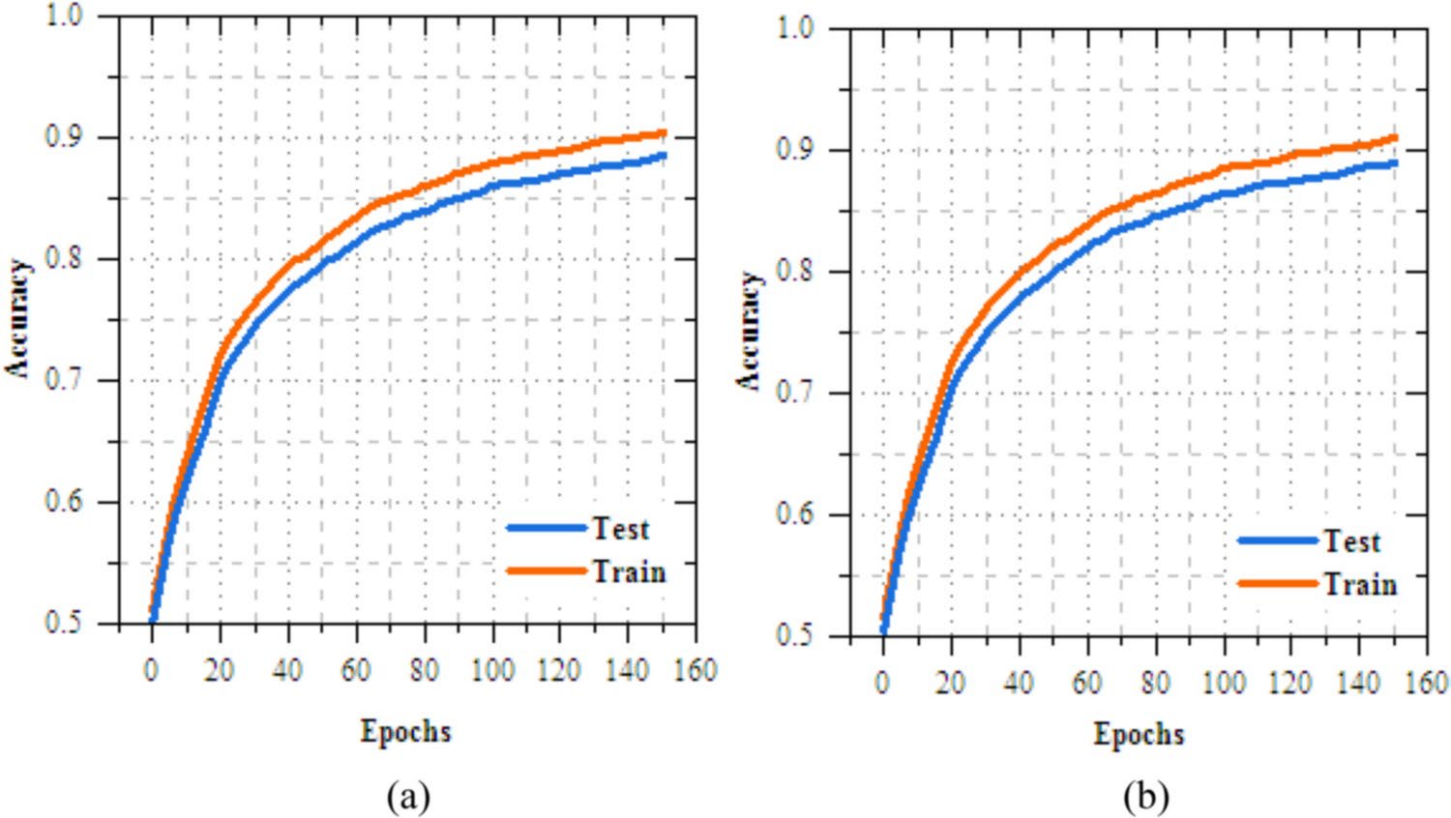
Training and testing accuracy of federated transfer learningmodel for skin disease diagnosison IID dataset (**a**) client-1, (**b**) client-2.

**Fig. 9:**
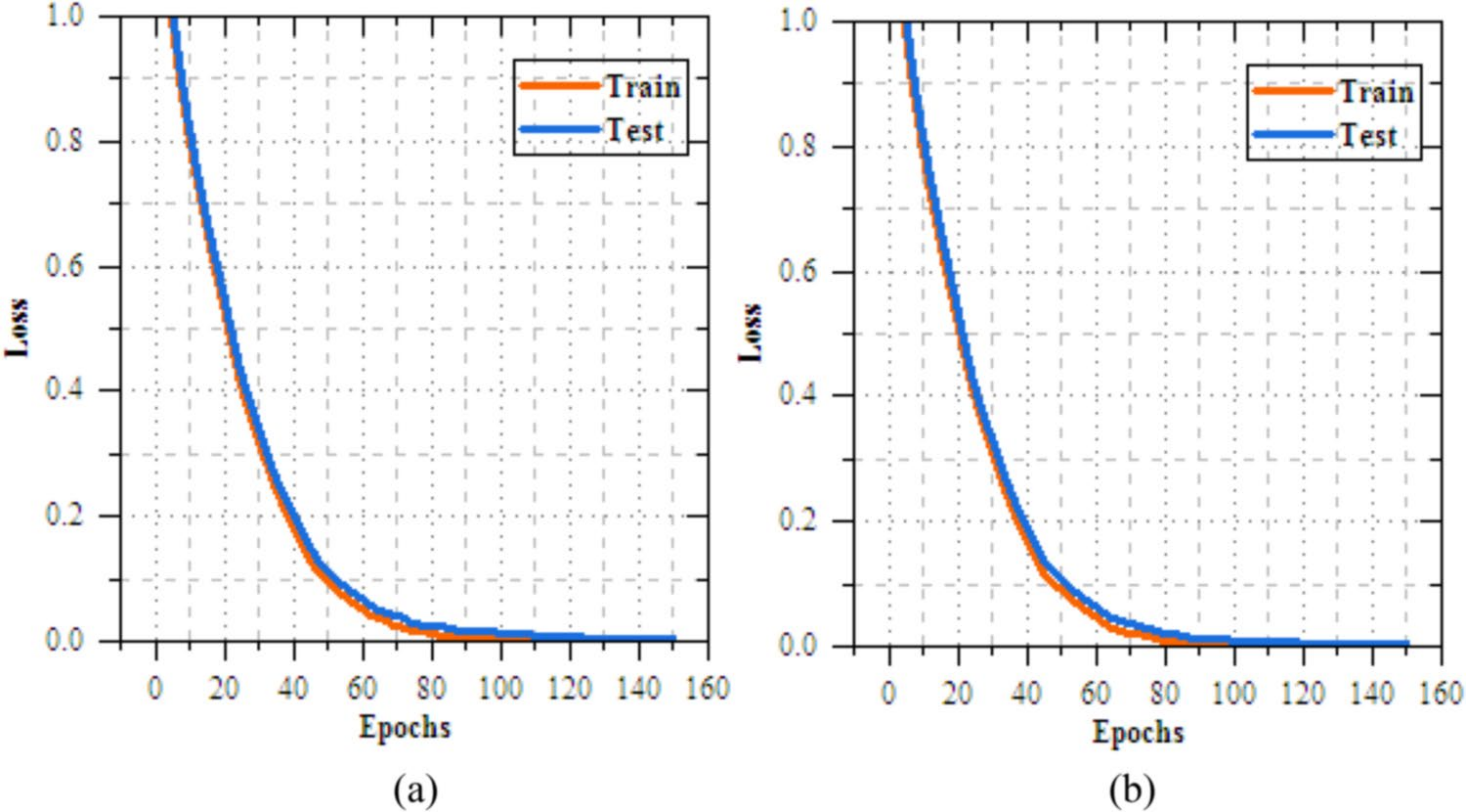
Training and testing loss of federated transfer learningmodel for skin disease diagnosison IID dataset (**a**) client-1, (**b**) client-2.

**Fig.10:**
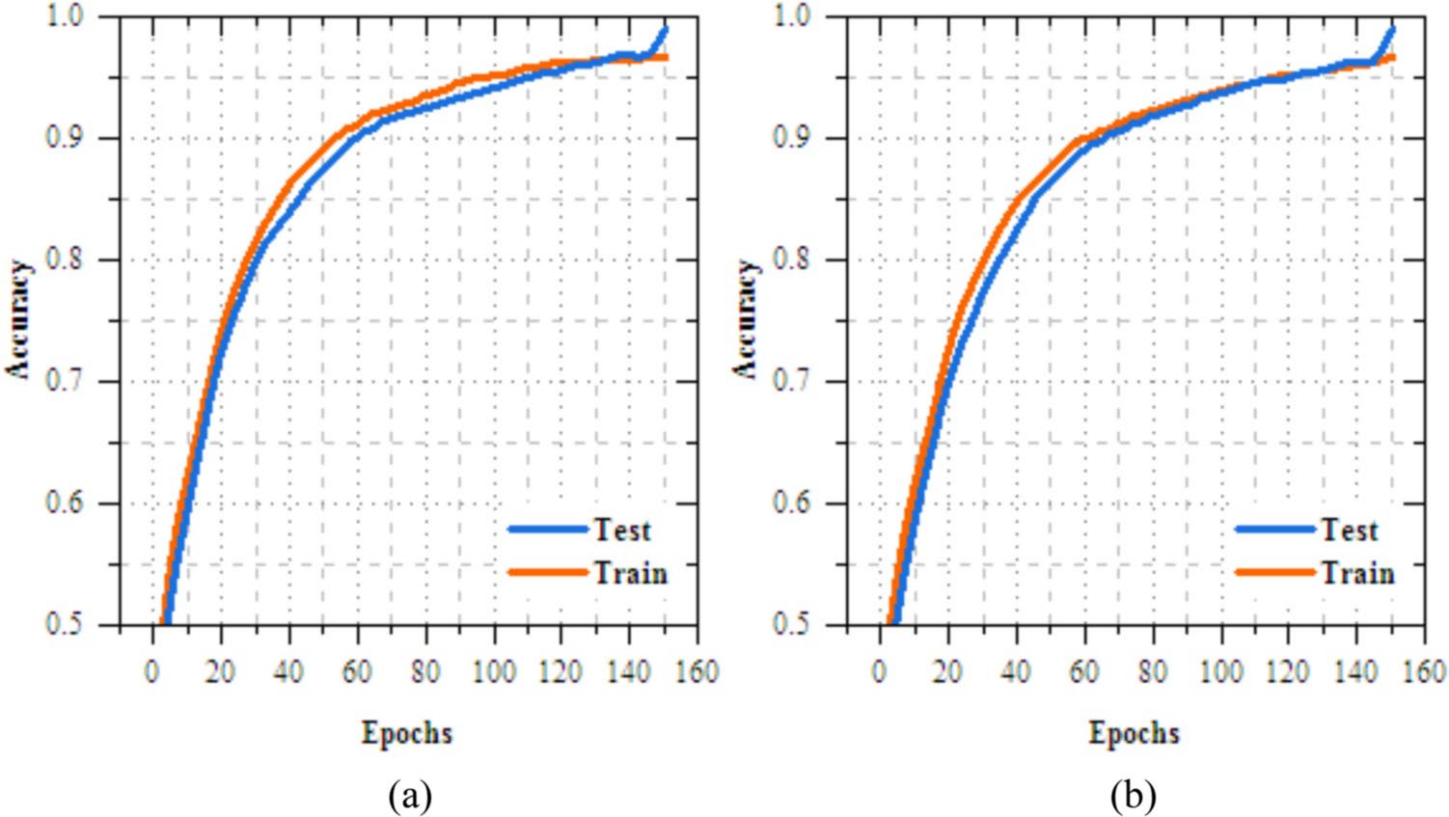
Training and testing accuracy of federated transfer learningmodel for skin disease diagnosison non-IID dataset (**a**) client-1, (**b**) client-2.

**Fig.11:**
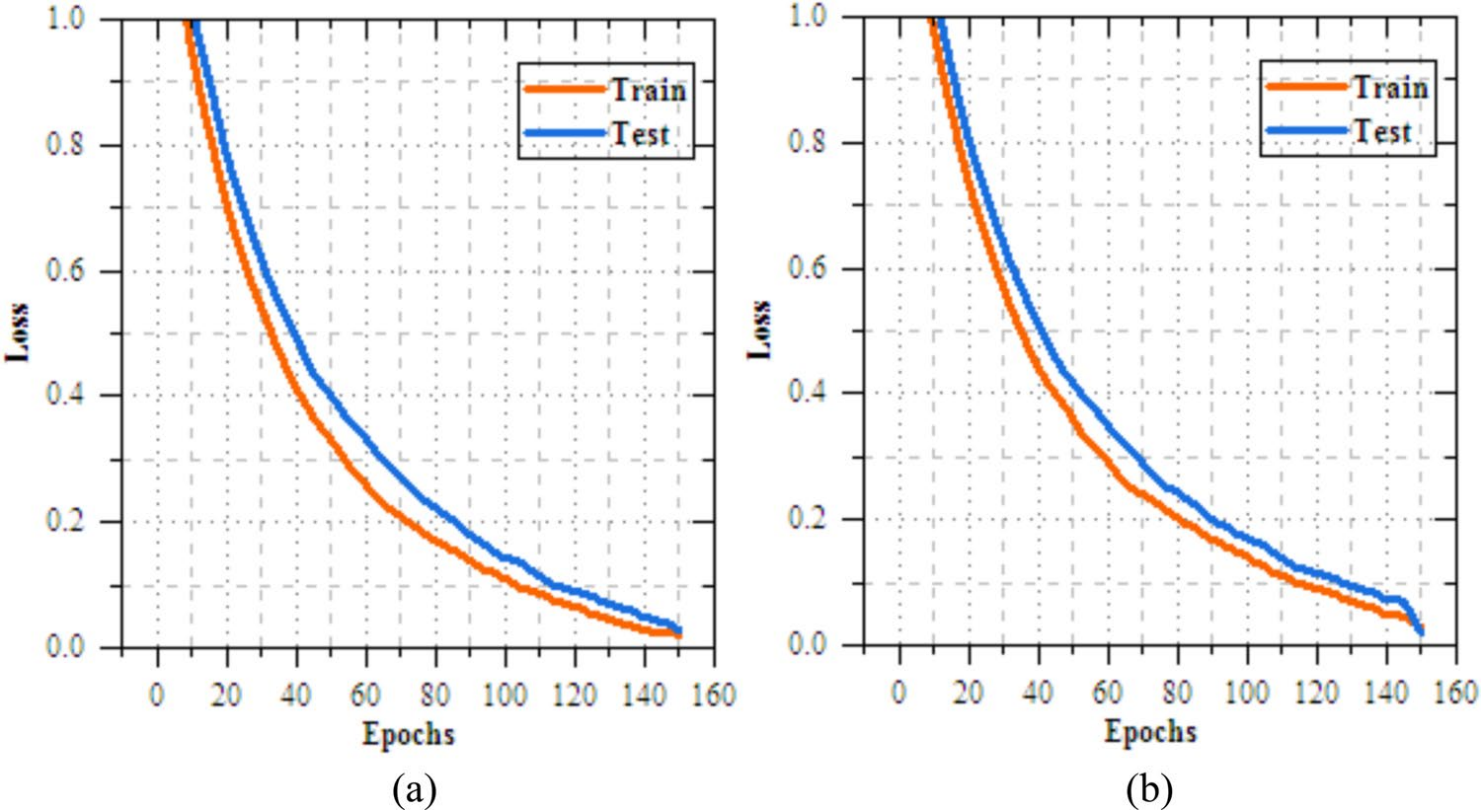
Training and testing loss of federated transfer learningmodel for skin disease diagnosis on non-IID dataset (**a**) client-1, (**b**) client-2.

The analysis of the federated transfer learning perfect for skin disease diagnosis demonstrates notable improvements in performance metrics across both Client 1 and Client 2 on the IID dataset. As highlighted in Section "Results analysis of feature extraction models on skin disease diagnosis", among the four transfer learning models evaluated, MobileNetV2 delivers the most effective results, achieved accuracy of 98.064%, making most suitable model for this experiment. As shown in Figs. 8 and 9, for Client 1, accuracy improves from 25.568% to 99.698%, while precision, recall, and F-measure increase from 17.82%, 19.856%, and 18.783% to 99.897%. On Non-IID datasets, as shown in Figs. 10 and 11, training outcomes exhibit performance over 25 epochs. The outcomes confirm the models’ effective learning and optimization, even with the testsmodeled by non-IID data circulations.

**Fig. 12:**
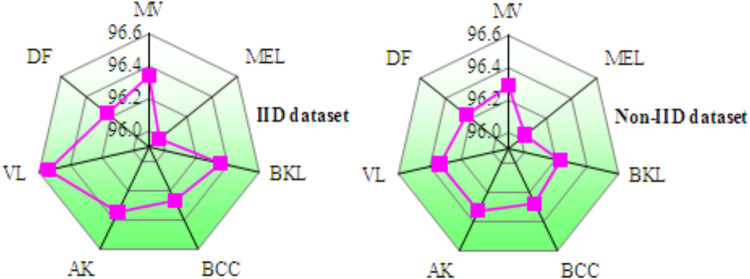
Accuracy of MobileNetV2 + FL + DNN for skin disease diagnosison IID and non-IID datasets.

Table 9 compares the presentation of federated transfer knowledgefor skin infectionfinding across IID and Non-IID datasets. During training, models on the Non-IID dataset demonstrate marginal improvements over IID data. Training accuracy rises from 96.428% to 96.573%, precision improves from 96.004% to 96.235%, recall increases from 96.108% to 96.389%, and F-measure advances from 96.048% to 96.298%. Additionally, the training loss decreases from 0.775 (IID) to 0.632 (Non-IID), indicates enhanced optimization on Non-IID data. In testing, the Non-IID dataset again outperforms the IID. Table 10 describes the class-wise accuracy of MobileNetV2 + FL + DNN model across tenfold cross-validation for both IID and Non-IID datasets shows highly consistent and stable performance in skin disease diagnosis. As shown in Fig. 12, the narrow clustering of average accuracy values between 95.9% and 96.5%, along with steady accuracy trends across all folds, confirms the robustness, reliability of the MobileNetV2 + FL + DNN perfect when used to both IID and Non-IID dataset. Table 11 summarizes the resource utilization metrics for the MobileNetV2 + FL + DNN model on both IID and Non-IID datasets for skin disease diagnosis, focusing on GPU memory, GPU process, CPU process, and virtual memory usage across two clients. The results confirm that resource utilization remains efficient and fairly stable between IID and Non-IID scenarios for this federated learning configuration.

**Table 9:** Results of federated transfer learning models for skin disease diagnosis.

Data	Data type	Values in (%)				Loss
		Accuracy	Precision	Recall	F-measure	
Training	IID	96.428	96.004	96.108	96.048	0.407
	Non-IID	96.573	96.235	96.389	96.298	0.485
Testing	IID	98.918	98.327	97.312	97.484	0.425
	Non-IID	99.063	98.558	98.796	98.674	0.493

**Table 10:** Class-wise accuracy of MobileNetV2 + FL + DNNfor skin disease diagnosis over k-fold cross validation.

Dataset	Class	K-fold cross validation									
		1	2	3	4	5	6	7	8	9	10
IID	MV	96.126	96.338	96.246	96.514	96.316	96.027	96.376	96.543	96.442	96.684
	MEL	95.785	95.971	95.909	96.13	96.013	95.856	96.23	95.973	96.118	95.844
	BKL	96.336	96.402	96.12	96.564	96.522	96.353	96.257	96.419	96.305	96.469
	BCC	96.034	96.217	96.105	96.362	96.499	96.199	96.252	96.342	96.46	96.528
	AK	96.233	96.371	96.453	96.152	96.297	96.291	96.345	96.526	96.445	96.226
	VL	96.541	96.692	96.463	96.509	96.745	96.459	96.292	96.677	96.526	96.698
	DF	96.004	96.122	96.242	96.318	96.038	96.221	96.161	96.283	96.429	96.511
Non-IID	MV	96.271	96.4	96.218	96.52	96.113	96.246	96.055	96.407	96.297	96.223
	MEL	95.917	96.031	96.029	96.248	95.89	96.015	96.162	96.31	95.873	95.991
	BKL	96.147	96.234	96.368	96.218	96.062	96.192	96.198	96.322	96.401	96.147
	BCC	96.263	96.195	96.358	96.486	96.409	96.34	96.191	96.185	96.266	96.393
	AK	96.326	96.433	96.253	96.522	96.185	96.32	96.376	96.491	96.345	96.149
	VL	96.437	96.309	96.223	96.121	96.492	96.254	96.386	96.295	96.511	96.434
	DF	96.003	96.124	96.21	96.319	96.421	96.198	96.255	96.367	96.155	96.079

**Table 11:** Resource metrics results of MobileNetV2 + FL + DNNfor skin disease diagnosis.

Client	Data Type	Minimum	Maximum	Average	Minimum	Maximum	Average
		GPU Memory Used (%)			GPU Process Used (%)		
Client 1	IID	28.360	30.280	29.320	31.050	32.120	31.585
Client 2		29.070	31.120	30.095	31.640	32.540	32.090
Client 1	Non-IID	28.580	30.850	29.715	29.940	31.890	30.915
Client 2		29.350	31.480	30.415	30.900	32.880	31.890
		CPU process used (%)			Virtual memory used (%)		
Client 1	IID	5.100	5.920	5.510	75.800	77.620	76.710
Client 2		5.050	5.840	5.445	75.960	77.250	76.605
Client 1	Non-IID	5.290	5.020	5.155	75.650	77.080	76.365
Client 2		5.420	5.100	5.260	75.950	77.400	76.675

### Results analysis of UNet + FL + DNN for skin disease diagnosis

**Fig. 13:**
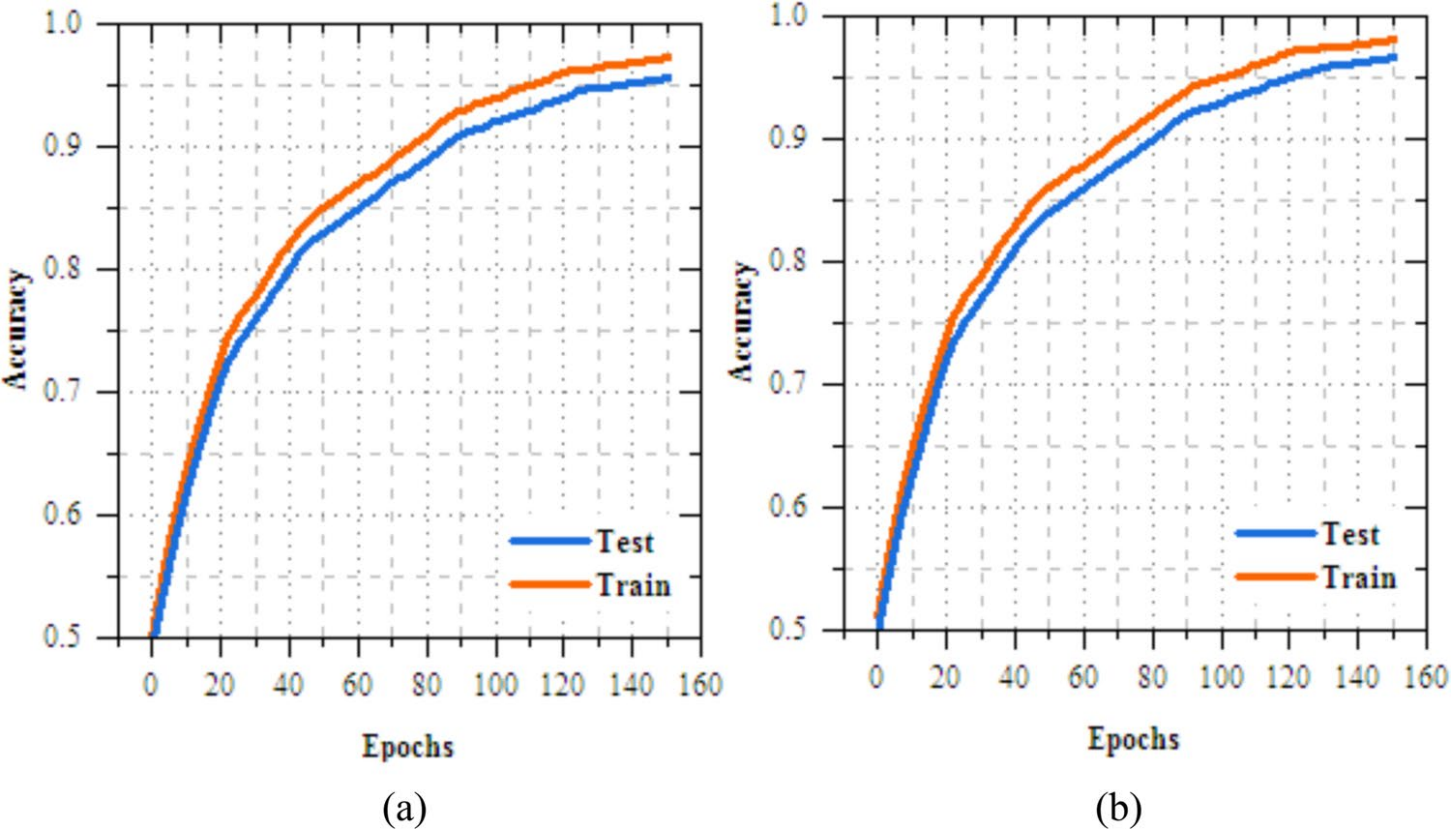
Training and testing accuracy of feature extraction with FL model for skin disease diagnosis on IID dataset (**a**) client-1, (**b**) client-2.

**Fig. 14:**
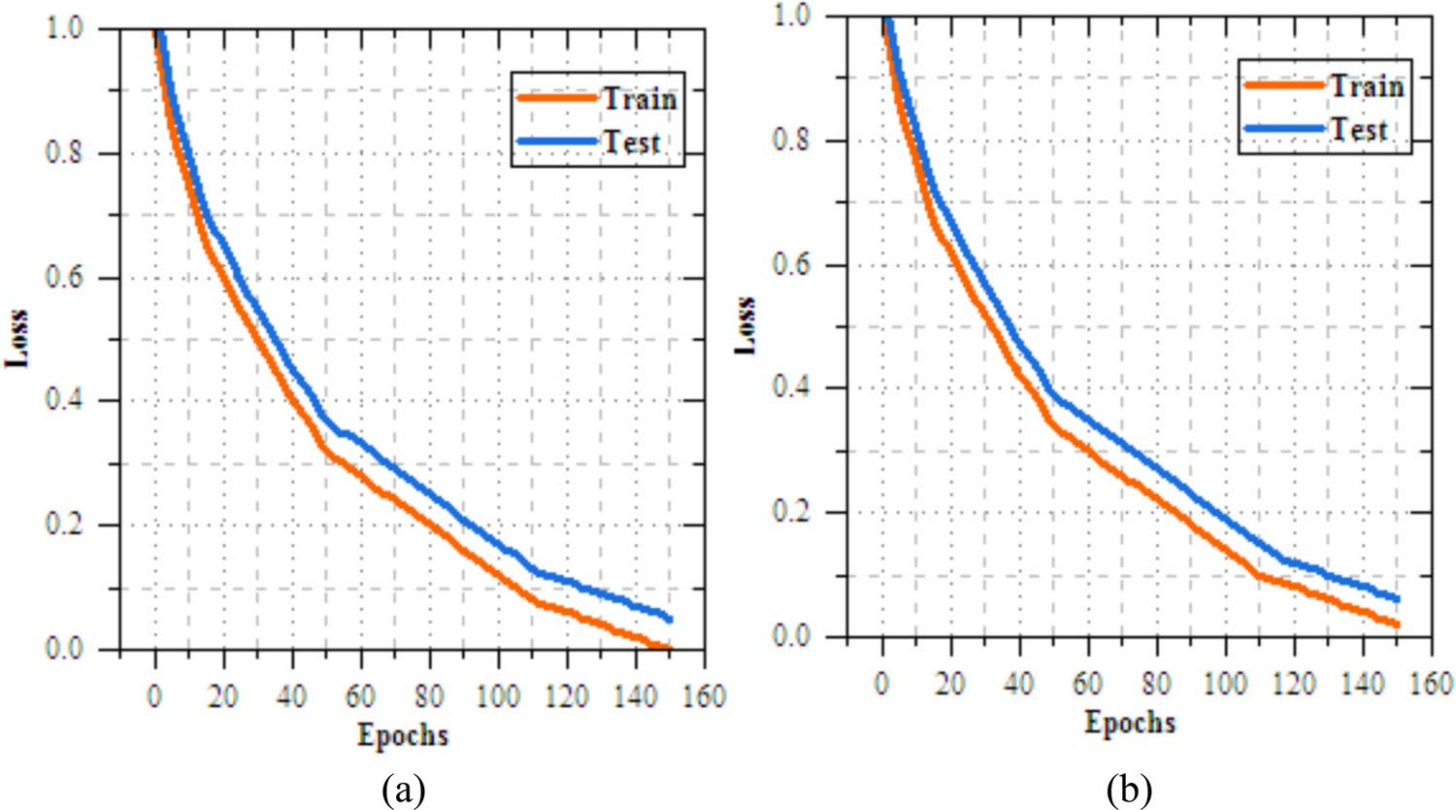
Training and testing loss of feature extraction with FL model for skin disease diagnosis on IID dataset (**a**) client-1, (**b**) client-2.

**Fig. 15:**
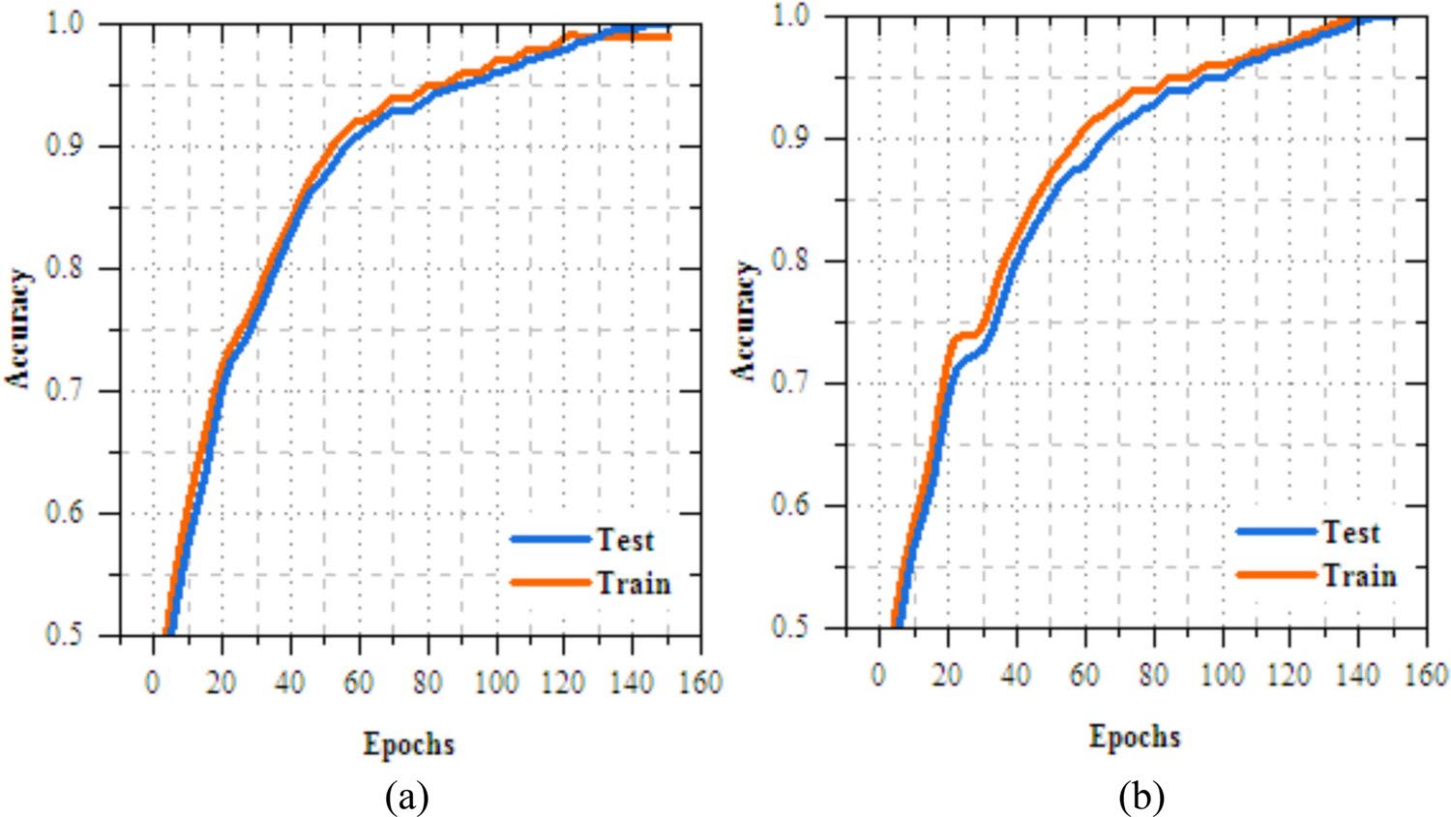
Training and testing accuracy of feature extraction with FL model for skin disease diagnosis on non-IID dataset (**a**) client-1, (**b**) client-2.

**Fig. 16:**
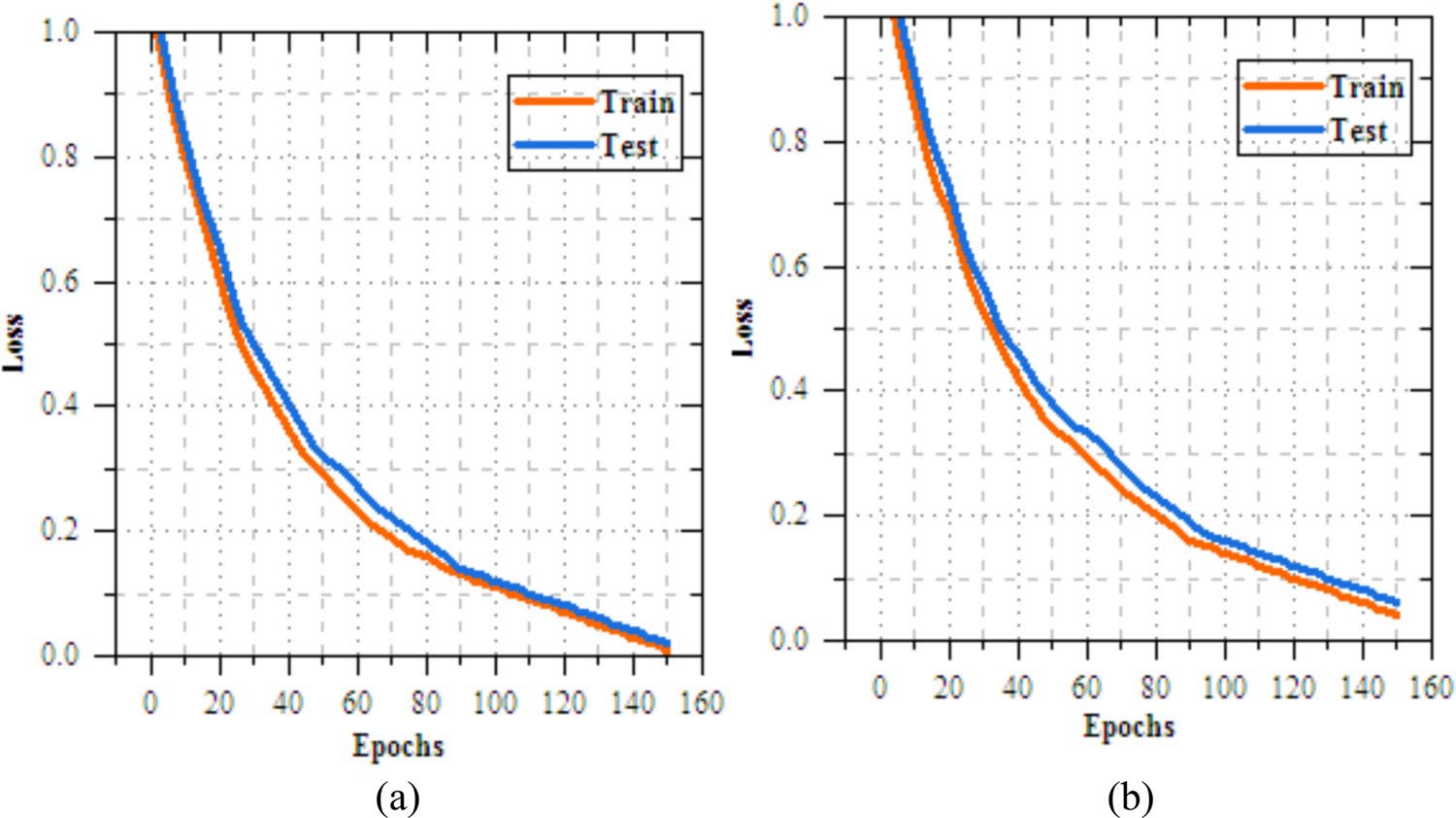
Training and testing loss of feature extraction with FLmodel for skin disease diagnosis on non-IID dataset (**a**) client-1, (**b**) client-2.

The UNet-based feature extraction model achieves a maximum training accuracy of 90.338% and testing accuracy of 83.854%. Figures 13 and 14 shows the training results for the UNet + FL model on the IID dataset for Client 1 and Client 2. Both clients show exceptional performance, with accuracy exceeding 99% by the final epoch. Figures. 15 and 16 show the training results of federated learning models for feature extraction on Non-IID datasets, comparing Client 1 and Client 2 over 25 epochs. Table 12 presents the effectiveness of the UNet + FL + DNN model for skin diseases diagnosis, comparing results with both IID and Non-IID datasets. During training, the model exhibits minor variations, with accuracy dropping from 99.514% for IID to 99.414% for Non-IID data. The loss is reduced for Non-IID data at 0.587 compared to 0.623 for IID. In the testing phase, the model shows superior performance with Non-IID data, with accuracy rising by 0.161% to 99.689%, precision increasing by 0.193% to 99.506%, recall by 0.144% to 99.441%, and F-measure by 0.171% to 99.473%.

**Table 12:** Results comparison of UNet + FL + DNN for skin disease diagnosis.

Data	Data type	Values in (%)				Loss
		Accuracy	Precision	Recall	F-measure	
Training	IID	99.514	99.382	99.291	99.315	0.398
	Non-IID	99.414	99.389	99.302	99.340	0.412
Testing	IID	99.528	99.313	99.297	99.302	0.415
	Non-IID	99.689	99.506	99.441	99.473	0.431

**Fig. 17:**
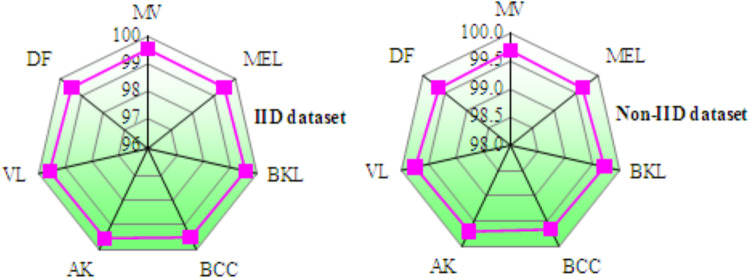
Accuracy of MobileNetV2 + FL + DNN for skin disease diagnosis on IID and non-IID datasets.

Table 13 depicts the class-wise accuracy performance of the proposed UNet + FL + DNN for skin disease diagnosis was evaluated using tenfold cross-validation on both IID and non-IID datasets. Figure 17 confirms that the MobileNetV2 + FL + DNN model maintained consistent and superior accuracy trends under both IID and non-IID distributions, displays the robustness of the FL framework for reliable skin disease diagnosis. Table 14 illustrates the resource utilization metrics for the UNet + FL + DNN model under IID and Non-IID data distributions in the context of skin disease diagnosis. Non-IID data introduces a mild increase in GPU memory, GPU process, and CPU process usage for both clients, with Client 1 generally experiencing slightly higher increases than Client 2 in most metrics. The virtual memory usage remains fairly consistent, indicating Non-IID data marginally raises computational demand and system resource utilization remains balanced and efficient in FL settings.

**Table 13:** Class-wise accuracy of UNet + FL + DNN for skin disease diagnosis over k-fold cross validation.

Dataset	Class	K-fold cross validation									
		1	2	3	4	5	6	7	8	9	10
IID	MV	99.512	99.543	99.528	99.567	99.499	99.535	99.522	99.549	99.515	99.541
	MEL	99.467	99.489	99.475	99.503	99.461	99.482	99.47	99.497	99.465	99.484
	BKL	99.548	99.572	99.559	99.585	99.541	99.563	99.551	99.577	99.546	99.569
	BCC	99.491	99.513	99.499	99.525	99.485	99.508	99.494	99.519	99.489	99.511
	AK	99.529	99.551	99.537	99.563	99.523	99.545	99.531	99.557	99.527	99.549
	VL	99.574	99.598	99.584	99.61	99.568	99.591	99.579	99.603	99.575	99.596
	DF	99.456	99.478	99.464	99.49	99.45	99.473	99.459	99.485	99.454	99.476
Non-IID	MV	99.673	99.705	99.689	99.721	99.661	99.697	99.682	99.709	99.675	99.701
	MEL	99.628	99.65	99.636	99.662	99.622	99.645	99.631	99.657	99.626	99.649
	BKL	99.709	99.733	99.718	99.745	99.703	99.726	99.712	99.738	99.707	99.73
	BCC	99.652	99.674	99.66	99.686	99.646	99.669	99.654	99.68	99.649	99.672
	AK	99.69	99.712	99.698	99.724	99.684	99.707	99.692	99.718	99.688	99.71
	VL	99.735	99.759	99.745	99.771	99.729	99.752	99.737	99.765	99.733	99.757
	DF	99.621	99.643	99.629	99.655	99.615	99.638	99.624	99.65	99.619	99.642

**Table 14:** Resource metrics results of UNet + FL + DNN for skin disease diagnosis.

Client	Data type	Minimum	Maximum	Average	Minimum	Maximum	Average
		GPU memory used (%)			GPU Process Used 9%)		
Client 1	IID	25.320	24.450	24.885	27.180	28.250	27.715
Client 2		25.410	23.010	24.210	27.450	29.020	28.235
Client 1	Non-IID	25.580	22.740	24.160	26.100	28.840	27.470
Client 2		25.760	23.180	24.470	26.820	29.450	28.135
		CPU process used (%)			Virtual memory used (%)		
Client 1	IID	3.180	4.850	4.015	73.240	75.120	74.180
Client 2		3.250	4.650	3.950	73.650	75.430	74.540
Client 1	Non-IID	3.350	4.020	3.685	72.980	75.020	74.000
Client 2		3.480	4.100	3.790	73.120	75.180	74.150

## Discussion

Table 15 presents a comparative analysis of accuracy, loss, and resource utilization for four model strategies in skin disease diagnosis: Strategy (a) as MobileNetV2, Strategy (b) as UNet, Strategy (c) as MobileNetV2 + FL + DNN, and Strategy (d) as UNet + FL + DNN. In terms of accuracy, integrating FL with DNN classification significantly improved performance. Under IID conditions, MobileNetV2 + FL + DNN achieved 99.063% accuracy, which is an 8.23% and 18.03% increase over MobileNetV2 and UNet, respectively. Inference speed was also faster in FL model, with UNet + FL + DNN achieving 28 ms (IID) and 29 ms (Non-IID), significantly quicker than MobileNetV2 (42 ms) and UNet (57 ms). In terms of model size, although federated models were slightly larger (24.6 MB for Strategy c and 27.1 MB for Strategy d), this increase is acceptable given their superior accuracy and efficiency. The resource consumption analysis further emphasizes the advantage of FL-based models. UNet + FL + DNN utilized the least GPU memory (24.548% IID and 24.315% Non-IID), compared to MobileNetV2 (76.2%) and UNet (69.5%), reflecting a reduction of 67.8% and 64.9%. GPU process utilization also decreased in FL models, with UNet + FL + DNN consuming only 27.975% (IID) and 27.803% (Non-IID), and MobileNetV2 + FL + DNN slightly higher. CPU process usage followed the same trend, with UNet + FL + DNN requiring the least at 3.983% (IID) and 3.738% (Non-IID), showed drop from MobileNetV2 and UNet. Virtual memory usage was similarly optimized in federated setups; with UNet + FL + DNN and MobileNetV2 + FL + DNN maintaining lower consumption levels than their standalone counterparts. The ANOVA results (Table 15) confirmed statistically significant differences (*p* < 0.001) in accuracy, loss, training time, and GPU-related metrics, indicating that the choice of strategy has a meaningful impact on performance. To further determine where these differences lie, Tukey’s HSD post-hoc analysis was applied. The post-hoc results revealed that Strategy (d) (UNet + FL + DNN) consistently outperformed Strategies (a), (b), and (c) with statistically significant higher accuracy and lower loss values. Similarly, both FL-integrated strategies (c and d) shows reduced resource usage (GPU/CPU/memory) compared to their non-federated counterparts (a and b), with strong statistical significance.

**Table 15:** Comparative analysis of accuracy, loss and resource consumption metrics for proposed models (strategy a, b, c and d) in skin disease diagnosis.

Resources	Strategy (a)	Strategy (b)	Strategy (c)		Strategy (d)		ANOVA (p)	Tukey’s HSD
	MobileNetV2	UNet	MobileNetV2 + FL + DNN		UNet + FL + DNN			
			IID	Non-IID	IID	Non-IID		
GPU memory (%)	76.200	69.500	29.708	30.065	24.548	24.315	< 0.001	(a),(b) > (c),(d)
Virtual memory (%)	81.865	77.635	76.658	76.520	74.360	74.075	< 0.05	(a) > (d)
GPU process (%)	69.300	62.120	31.838	31.403	27.975	27.803	< 0.001	(a),(b) > (c),(d)
CPU process (%)	11.450	8.715	5.478	5.208	3.983	3.738	< 0.001	(a),(b) > (c),(d)
Training time (s)	1380	1560	1020	1065	945	990	< 0.001	(b) > all, (d) < all
Model size (MB)	16.700	19.300	24.600	24.600	27.100	27.100	< 0.01	(d) > (a)
Inference speed (ms)	42.000	57.000	31.000	33.000	28.000	29.000	< 0.001	(b) > all, (d) < all
Mean accuracy (%)	91.53	83.854	98.918	99.063	99.528	99.689	< 0.001	(d) > (a),(b),(c)
Mean loss	0.502	0.612	0.425	0.493	0.415	0.431	< 0.001	(b) > (d), (a) > (c),(d)

In real-world clinical settings, computational resource efficiency plays a crucial role in determining the deployability of AI models, especially in resource-constrained environments such as small clinics or mobile diagnostic units. From the comparative analysis (Table 15), observe that traditional models like MobileNetV2 and UNet require higher GPU memory and longer training times, which not be feasible for on-site training or rapid inference. UNet + FL + DNN, in particular, requires only 24.548% GPU memory and 945 s of training time, while offering the fastest inference speed of 28 ms. FL-based model offer enhanced data privacy, aligning with regulatory frameworks like HIPAA and GDPR, which is critical for clinical use. The slightly larger model sizes (27.1 MB for UNet + FL + DNN) are still manageable on modern edge devices and embedded systems, making these models highly practical for deployment in decentralized clinical infrastructures without compromising diagnostic accuracy.

## Conclusion

A privacy-preserving FL framework was proposed for skin disease diagnosis, evaluated through four strategic approaches: strategy (a) employed MobileNetV2 with transfer learning and DNN classification, strategy (b) utilized UNet for feature extraction followed by DNN classification, strategy (c) integrated FL with MobileNetV2 and DNN, and strategy (d) combined UNet-based feature extraction with FL and DNN classification to maintain data decentralization while enhancing diagnostic accuracy. Both IID and Non-IID datasets were used for comprehensive assessment. From the results, strategy (d) achieved the highest diagnostic accuracy of 99.689% (IID), surpassing MobileNetV2 by 8.16% and UNet by 15.835%. It also recorded the lowest loss of 0.415 (IID), representing a 17.32% reduction compared to MobileNetV2 and 32.21% decrease relative to UNet. Strategy (c) delivered performance with 98.918% accuracy (IID) and a loss of 0.425, improving substantially over both baseline models though marginally lower than strategy (d). In terms of resource consumption, strategy (d) required 24.548% GPU memory (IID) and 27.975% GPU process, which were significantly lower than MobileNetV2 and UNet. Strategy (c) followed closely with 29.708% GPU memory and 31.838% GPU process. Similar trends were noted for CPU process and virtual memory usage, where federated models consumed fewer resources while achieving higher accuracy and lower loss values. When comparing strategy d to strategy c, the former outperformed with 0.771% higher accuracy and 2.35% lower loss which confirms that incorporating feature extraction through UNet prior to federated optimization results in superior classification outcomes compared to direct transfer learning fine-tuning in a federated environment. The proposed strategy (d) model effectively combines high diagnostic accuracy with strong data privacy safeguards. It ensures reliable, scalable, and privacy-preserving skin disease detection across both IID and Non-IID data distributions. These capabilities position the model as a robust AI-driven dermatological solution, highly suitable for real-world telemedicine and remote healthcare applications.

## Data Availability

The data supporting the findings of this study are available from the corresponding author upon reasonable request. We confirm that all necessary steps were taken to ensure the privacy and confidentiality of the data used in this research. The HAM10000 dataset is publicly available and does not contain any personally identifiable information. Additionally, the proposed federated learning approach inherently protects data privacy by keeping the raw data localized to each client device. No identifying information or individual patient details were included in the manuscript.https://www.kaggle.com/datasets/vrindaat/ham10000-dataset

## References

[1] Papadopoulos, L. & Walker, C. *Understanding skin problems: acne, eczema, psoriasis and related conditions* (John Wiley & Sons, 2003).

[2] Ou, M., Xue, Y., Qin, Y., & Zhang, X. (2024). Experience and caring needs of patients with psoriasis: A qualitative meta‐synthesis. J. Clin. Nurs. 10.1111/jocn.17146 38616578

[3] Nearchou, F., &Flinn, C. (2024). The Impact of COVID-19 on Children and Adolescents with Chronic Illness. The COVID-19 Aftermath: Volume I: Ongoing Challenges, 385–399. 10.1007/978-3-031-61939-7_22 39283439

[4] Li Pomi, F. et al. Artificial intelligence: a snapshot of its application in chronic inflammatory and autoimmune skin diseases. *Life* **14**(4), 516 (2024). 38672786 10.3390/life14040516 PMC11051135

[5] Vayadande, K. (2024). Innovative approaches for skin disease identification in machine learning: A comprehensive study. Oral Oncol. Reports 100365.

[6] Brown, M. et al. Topically applied therapies for the treatment of skin disease: past, present, and future. *Pharmacol. Rev.* **76**(5), 689–790 (2024). 38914467 10.1124/pharmrev.123.000549

[7] Singh, J., Sandhu, J. K. & Kumar, Y. An analysis of detection and diagnosis of different classes of skin diseases using artificial intelligence-based learning approaches with hyper parameters. *Arch. Computat. Method. Eng.* **31**(2), 1051–1078 (2024).

[8] Dang, T. L. P., Sadreddin, A. & Ahuja, S. Readily available technologies in low-resource communities: a review and synthesis. *Inf. Technol. Dev.* **30**(1), 132–172 (2024).

[9] El-Saleh, A. A., Sheikh, A. M., Albreem, M. A., Honnurvali, M. S. (2024). The Internet of Medical Things (IoMT): opportunities and challenges. Wireless Networks 1–18.

[10] Rosário, A. T., &Rosário, I. T. (2024). Telemedicine Platforms and Telemedicine Systems in Patient Satisfaction. In Improving Security, Privacy, and Connectivity Among Telemedicine Platforms (pp. 119–151). IGI Global.

[11] Sitaraman, S. R., Alagarsundaram, P., Kumar, V. & Kurniadi, D. Accurate skin disease detection with K-nearest neighbors and CAM in IoMT-enabled diagnostic solutions. *Chin. Tradi. Med. J.* **7**(3), 5–17 (2024).

[12] Ahmed, S. F. et al. Insights into internet of medical things (IoMT): Data fusion, security issues and potential solutions. *Inform. Fusion* **102**, 102060 (2024).

[13] Khatiwada, P., Yang, B., Lin, J. C. & Blobel, B. Patient-generated health data (PGHD): Understanding, requirements, challenges, and existing techniques for data security and privacy. *J. Personal. Med.* **14**(3), 282 (2024). 10.3390/jpm14030282 PMC10971637 38541024

[14] Kissi, J. et al. Healthcare professionals’ perception on emergence of security threat using digital health technologies in healthcare delivery. *Digit. Health* **10**, 20552076241260384 (2024). 38868369 10.1177/20552076241260385 PMC11168049

[15] Alsamhi, S. H., Myrzashova, R., Hawbani, A., Kumar, S., Srivastava, S., Zhao, L., Curry, E. (2024). Federated learning meets blockchain in decentralized data-sharing: Healthcare use case. IEEE Internet of Things J.

[16] Williamson, S. M. & Prybutok, V. Balancing privacy and progress: a review of privacy challenges, systemic oversight, and patient perceptions in AI-driven healthcare. *Appl. Sci.* **14**(2), 675 (2024).

[17] Albalawi, E., TR, M., Thakur, A., Kumar, V. V., Gupta, M., Khan, S. B., Almusharraf, A. (2024). Integrated approach of federated learning with transfer learning for classification and diagnosis of brain tumor. BMC Med. Imag. 24(1), 110. 10.1186/s12880-024-01261-0 PMC11097560 38750436

[18] Schreiber, R., Koppel, R. & Kaplan, B. What do we mean by sharing of patient Data? DaSH: A data sharing hierarchy of privacy and ethical challenges. *Appl. Clin. Inform.* **15**(05), 833–841 (2024). 39053616 10.1055/a-2373-3291 PMC11483170

[19] Vinudevi, G., Vijayaragavan, S. P., Karthik, B. (2024). Transfer Learning Approaches for Colorectal Tumour Detection on Adapting Pre-Trained Models to Diverse Medical Imaging Datasets. In Optimizing Intelligent Systems for Cross-Industry Application (pp. 411–432). IGI Global.

[20] Choudhry, I. A. et al. Privacy-preserving AI for early diagnosis of thoracic diseases using IoTs: A federated learning approach with multi-headed self-attention for facilitating cross-institutional study. *Internet of Things* **27**, 101296 (2024).

[21] Divya, N. D. & Sharma, G. Convolutional neural network (CNN) and federated learning-based privacy preserving approach for skin disease classification. *The J. Supercomput.* **80**(16), 24559–24577 (2024).

[22] Kareem, A. (2024). A privacy-preserving approach to effectively utilise distributed data for medical disease detection. 10.3390/bioengineering11040340 PMC11048296 38671762

[23] Barnawi, A., Chhikara, P., Tekchandani, R., Kumar, N., &Alzahrani, B. (2024). A Differentially Privacy Assisted Federated Learning Scheme to Preserve Data Privacy for IoMT Applications. IEEE Trans. Network Service Manag.

[24] Aminifar, A., Shokri, M., &Aminifar, A. (2024). Privacy-Preserving Edge Federated Learning for Intelligent Mobile-Health Systems. arXiv preprint arXiv:2405.05611.

[25] Zhang, Z., Ming, Y. & Wang, Y. A federated transfer learning approach for surface electromyographic hand gesture recognition with emphasis on privacy preservation. *Eng. Appl. Artif. Intell.* **136**, 108952 (2024).

[26] Kumar, R., Bernard, C. M., Ullah, A., Khan, R. U., Kumar, J., Kulevome, D. K., Zeng, S. (2024). Privacy-preserving blockchain-based federated learning for brain tumor segmentation. Comput. Biol. Med. 108646. 10.1016/j.compbiomed.2024.108646 38824788

[27] Alahmadi, A., Khan, H. A., Shafiq, G., Ahmed, J., Ali, B., Javed, M. A., Alahmadi, A. H. (2024). A privacy-preserved IoMT-based mental stress detection framework with federated learning. J. Supercomput. 80(8), 10255–10274.

[28] Jiang, X., Zhang, J. & Zhang, L. Fedradar: Federated multi-task transfer learning for radar-based internet of medical things. *IEEE Trans. Netw. Serv. Manage.* **20**(2), 1459–1469 (2023).

[29] Nam, B. J. (2023). Skin Disease Classification Using Privacy-Preserving Federated Learning. Int. J. High School Res. 5(1).

[30] Wang, W., Li, X., Qiu, X., Zhang, X., Brusic, V., Zhao, J. (2023). A privacy preserving framework for federated learning in smart healthcare systems. Inform. Process. Manag. 60(1), 103167.

[31] Nam, B.J., 2023. Skin Disease Classification Using Privacy-Preserving Federated Learning. Int. J. High School Res. 5(1).

[32] Hossen, M. N. et al. Federated machine learning for detection of skin diseases and enhancement of internet of medical things (IoMT) security. *IEEE J. Biomed. Health Inform.* **27**(2), 835–841 (2022). 10.1109/JBHI.2022.3149288 35133971

[33] Gupta, M., Kumar, M. & Gupta, Y. A blockchain-empowered federated learning-based framework for data privacy in lung disease detection system. *Comput. Hum. Behav.* **158**, 108302 (2024).

[34] Lei, B. et al. Hybrid federated learning with brain-region attention network for multi-center Alzheimer’s disease detection. *Pattern Recogn.* **153**, 110423 (2024).

[35] Zhou, L., Wang, M. and Zhou, N., 2024. Distributed federated learning-based deep learning model for privacy mri brain tumor detection. arXiv preprint arXiv:2404.10026.

[36] Mitrovska, A., Safari, P., Ritter, K., Shariati, B. & Fischer, J. K. Secure federated learning for Alzheimer’s disease detection. *Front. Aging Neurosci.* **16**, 1324032 (2024). 38515517 10.3389/fnagi.2024.1324032 PMC10954782

[37] Vats, S., Kukreja, V. and Mehta, S., 2024, March. Tea Leaf Disease Detection: Federated Learning CNN Used for Accurate Severity Analysis. In 2024 IEEE International Conference on Interdisciplinary Approaches in Technology and Management for Social Innovation (IATMSI) (Vol. 2, pp. 1–6). IEEE.

[38] Chhikara, J., Goel, N. and Rathee, N., 2024, October. A critical analysis of transfer learning models for computer vision tasks. In AIP Conference Proceedings (Vol. 3209, No. 1). AIP Publishing.

[39] Hussain, A. & Aslam, A. Ensemble-based approach using inception V2, VGG-16, and Xception convolutional neural networks for surface cracks detection. *J. Appl. Res. Technol.* **22**(4), 586–598 (2024).

[40] Vayadande, K., 2024. Innovative approaches for skin disease identification in machine learning: A comprehensive study. Oral Oncology Reports, p.100365.

[41] Adebiyi, A., Abdalnabi, N., Smith, E.H., Hirner, J., Simoes, E.J., Becevic, M. and Rao, P., 2024. Accurate Skin Lesion Classification Using Multimodal Learning on the HAM10000 Dataset. medRxiv, pp.2024–05.

